# Revealing community dynamics in polymicrobial infections through a quantitative framework

**DOI:** 10.1093/ismeco/ycag061

**Published:** 2026-03-13

**Authors:** Aanuoluwa E Adekoya, Tyler E Boggs, Carolyn B Ibberson

**Affiliations:** Department of Microbiology, University of Tennessee, Knoxville, TN 37996, United States; Department of Microbiology, University of Tennessee, Knoxville, TN 37996, United States; Department of Microbiology, University of Tennessee, Knoxville, TN 37996, United States

**Keywords:** metatranscriptomics, microbe-microbe interaction, polymicrobial infection, chronic wounds, microbial ecology, bacterial pathogenesis

## Abstract

Laboratory models provide tractable, reproducible systems that have long served as foundational tools in microbiology. However, the extent to which these models accurately mimic the biological environments they represent remains poorly understood. A quantitative framework was recently introduced to assess how well laboratory models capture microbial physiology in situ. However, applications of this framework have been limited to characterizing the physiology of a single species in human infections, leaving a gap in our understanding of overall microbial community physiology in polymicrobial contexts. Here, we extended this framework to evaluate the accuracy of laboratory model systems in capturing community-level functions in polymicrobial infection. As a proof of concept, we applied the extended framework to a polymicrobial model of human chronic wound (CW) infection. CWs harbor metabolically diverse bacterial species that engage in a range of microbe-microbe interactions, ultimately impacting community dynamics and disease progression. However, studies on the mechanistic drivers of chronic wound infection have relied on single species or pairwise approaches. Here, we demonstrate that our adapted framework can be used to develop accurate polymicrobial models. Further, we demonstrate that this extended framework can evaluate the occurrence of known microbe-microbe interactions. Building on our prior work in large-scale metagenomic and metatranscriptomic analysis, we propose a highly accurate 6-member synthetic bacterial community model i.e. representative of the taxonomic and functional complexity of human CW infections. This approach will support the development of ecologically relevant polymicrobial models and better treatment strategies.

## Introduction

From the start, microbiology has explored microbial dynamics across diverse environments, including within the human body, where direct access is limited. This progress has been largely enabled by laboratory models that offer tractable systems that can be reproduced and manipulated for in-depth investigation [1–3]. While experimental models have proven invaluable for understanding microbial infection physiology, how accurately they mimic the native biological environment they represent is poorly understood. Recognizing the importance of rational laboratory model selection, a quantitative accuracy score framework using RNA sequencing data was proposed and used to improve two experimental model systems of infection [1, 4, 5]. However, despite the significance of this framework, its application has been limited to characterizing the physiology of a single species in human infection, leaving a gap in our understanding of microbial physiology and community dynamics in polymicrobial contexts.

To address this knowledge gap, we adapted this framework to evaluate the accuracy of overall microbial community function in polymicrobial environments. We used chronic wound infections as a test case, as they have multiple features that make them an ideal study system for this approach including a highly relevant polymicrobial environment, a relatively simple community composition [6–9], accessibility for in situ sampling, previously defined interactions between microbial community members [10–15], and measurable outcomes of infection. Chronic wounds (CWs) are wounds that fail to heal over a prolonged period (typically beyond 6 weeks) [16–19]. These wounds are characterized by damaged tissues that release extracellular matrix proteins that serve as nutrients and adhesion sites for bacterial colonization [17, 20], and are often hypoxic allowing for growth of microorganisms with diverse metabolic capacities [9, 17]. These communities establish a range of interactions, which has been shown to impact their survival and overall disease progression [10, 21–25]. However, research on microbial drivers of chronic wound infection has primarily focused on single species or pairwise interactions that do not capture the complexity of microbial communities in wound environments. This knowledge gap reduces the extent to which we can compare laboratory findings to clinical outcomes.

To address this limitation, we quantified the accuracy of microbial community function in experimental CW models compared to human CW infection and evaluated how modifications to community composition and host environment impacted experimental model accuracy. We used our community accuracy score framework to assess if known microbe-microbe interactions observed in experimental models are occurring in the infection environment. Further, we leveraged these findings to describe a highly accurate 6-member synthetic wound model system that better reflects the taxonomic and functional complexity of bacterial infection in human CWs. The use of accurate model systems will allow for improved mechanistic studies that capture microbial dynamics in human CW and allow for better correlations of laboratory findings and clinical outcomes.

## Materials and Methods

Detailed methodology is provided in the supplementary material.

## Results

### Adapting the quantitative framework to a polymicrobial context

**Figure 1:**
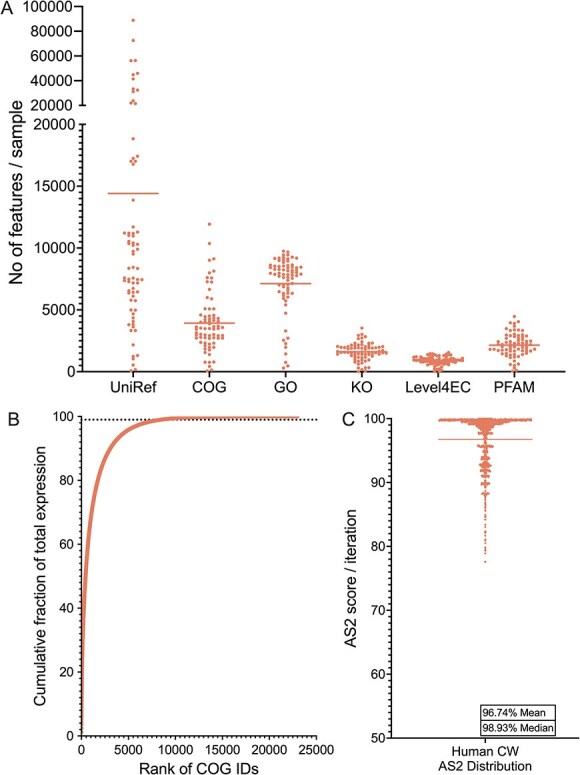
Distribution of functions and accuracy scores in human chronic wound metatranscriptomes. (A) Distribution of the different functional categories in human CW samples. Lines at means (UniRef 14 408, COG 3924, GO 7121, KEGG 1607, LevelEC 890, PFAM 2141). Each point represents the number of features identified in each human chronic wound sample for each of the annotation schemes. (B) Contribution of each COG ID to the total expression across the 66 human CW samples. COG IDs are ordered by their cumulative expression levels. Line at 8486 indicates the point where 99% of the total expression is captured. (C) Distribution of the accuracy scores of the subsampled human chronic wound samples across 1200 iterations, line at mean (96.74%).

We aimed to assess if a previously described accuracy score framework [1, 4, 5] could be applied to a polymicrobial context. This approach uses gene expression data to compare bacterial physiology in an experimental model to expression in the actual environment of interest. That is, gene or functional expression in the in situ environment of interest is considered as the benchmark for comparison of the expression of the same genes or functions in any experimental model. However, the application of this framework has been limited to individual bacterial species in human infection [1, 4, 5]. Therefore, we first sought to adapt this framework to capture overall microbial community function. In this framework, gene expression in the environment of interest, here human CW infection, is used as the benchmark. We pulled 72 CW metatranscriptomic samples (Table S3) from three previously published studies [26–28] to serve as this benchmark dataset. We used HUMANn4 [29] to assign prokaryotic reads to UniRef90 gene families as in our previous work [6], which ranged in expression from 68 copies per million (CPM) to 88 879 CPM (mean of 14 408 CPM; Dataset S1A) across the 72 CW samples. Due to the high functional and taxonomic specificity of UniRef90 IDs [30], we aimed to collapse this data to broader functional categories such that gene families with similar functions are grouped together. However, several functional grouping schemes exist, each with varying tradeoffs. Therefore, we evaluated Gene Ontology (GO terms—mean 7121, range 296–9745), Cluster Orthologous Genes (EggNOG/COG—mean 3924, range 52–11 933), KEGG Orthology (KO terms mean 1607, range 14–3532), Level 4 Enzyme Classification (Level4EC mean 890, range 16–1550) and Protein Family (PFAM—mean 2141, range 42–4461) to compare these annotation approaches (Fig. 1A). We found the EggNOG/COG classification provided the best coverage with reduced duplication compared to the other approaches (Fig. 1A and Dataset S1B), and used COG classified functional data for the rest of our analyses.

Using the COG/EggNOG ID classification we observed an average of 3924 COG IDs per sample, however six samples had poor coverage, defined as expression of fewer than 1000 COG IDs and fewer than 2000 UniRef90 IDs (Fig. S1B and Dataset S1A). We removed those samples from subsequent analyses and identified rarely expressed COG IDs across the remaining 66 human CW samples to minimize impacts of sparse data. Each COG ID was ranked by expression and prevalence, which revealed that while there were 22 986 distinct COG IDs across our dataset (Fig. 1B and Dataset S1C), only 8486 COG IDs accounted for over 99% of the cumulative expression, indicating numerous rarely expressed COG IDs (Fig. S1C and Dataset S1C). Specifically, over 14 000 COG IDs had a cumulative abundance of <1 CPM when summed across all samples in which they appeared and 40% of these were detected in only one sample. Further, these rarely expressed functions accounted for less than 5.5% of the expressed functions in any given human CW sample (Fig. S1D).

We used these 8486 COG IDs to calculate the accuracy score (AS_2_) of the human CW samples (Table S3) using a leave-two-out cross-validation approach. In each iteration, two samples were excluded and compared to the remaining dataset, and this was repeated for every possible pair, totaling over 1200 iterations. The accuracy score is then defined as the percentage of expressed functions (COG IDs) in the excluded samples that fell within ±2 standard deviations from the remaining dataset (AS_2_). We found while the AS_2_ across the leave-two-out iterations were between 77.59% and 100%, the distribution was negatively skewed, indicating most iterations yielded high accuracy scores with a mean of 96.74% (95% CI: 96.51–96.97) (Fig. 1C). Together, this demonstrated the 66 human CW samples have high similarity, irrespective of the patient characteristics or study experimental design. We used this mean (96.74%) as the benchmark for evaluating how well the in vitro and in vivo *models* developed in this study captured microbial community physiology in human CW infections.

### The quantitative framework can be used to assess microbial dynamics in a polymicrobial model

**Figure 2:**
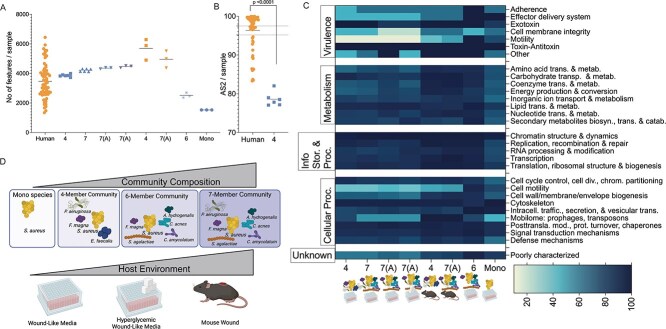
Community composition and host environment are key drivers of accuracy scores. (A) Distribution of COG IDs in all samples evaluated for accuracy score. Line at mean (human 3461, 4-member WLM 3860, 7-member WLM 4218, 7-member altered WLM 4359, 7-member altered hyperglycemic WLM 4462, 4-member murine 5694, 7-member murine 4961, 6-member WLM 2511, *S. aureus* mono-culture WLM 1531). (B) The AS_2_ of the 4-member WLM model is significantly different from the human CW samples AS_2_ (*P* < .0001, Mann–Whitney test). Dotted lines on 95% CI of human CW AS2 mean (lower = 95.18; upper = 97.49). (C) Heatmap of the accuracy scores of each subcategory. in the samples evaluated with the overall accuracy scores being 4-member WLM 77.98%, 7-member WLM 78.00%, 7-member altered WLM 82.02%, 7-member altered hyperglycemic WLM 77.62%, 4-member murine 89.70%, 7-member murine 90.78%, 6-member WLM 96.27%, *S. aureus* mono-culture 89.80%. (D) Two axes that can be manipulated iteratively to improve the overall accuracy of our experimental model system. Abbreviations: 4 = 4-member, 7 = 7-member, 7(A) = 7-member with altered inoculum, 6 = 6-member, Mono = *S. aureus* monoculture in any condition. Figure 2D was generated with Biorender.

To validate this adapted framework, we employed a previously published 4-member polymicrobial laboratory CW model [31]. We seeded *Staphylococcus aureus, Pseudomonas aeruginosa, Enterococcus faecalis* and *F. magna* into wound-like media (WLM) [31–33], a well validated in vitro model of human CW infection nutritionally similar to wound fluid [21], comprised of blood, plasma, and Bolton broth that allows for spatial structuring of the community due to coagulation by *S. aureus* for growth. To ensure that our experimental model would allow for growth of anaerobes, which have been shown to be abundant in CW and associated with poor healing [6, 8, 34, 35], we measured oxygen concentration over time using a 25 μM oxygen microsensor probe (OX-25 Unisense microprofiling system) (Fig. S2A) and observed there was a greater than 90% reduction in oxygen concentration to <15 μmol/L in each culture well within the first 24 h (Fig. S2B) consistent with the reduced oxygen levels observed during the progression of chronic wounds [21, 36–38]. At 48 h post inoculation, we obtained samples for transcriptomic data. We used the annotation approach highlighted above to determine the accuracy score of this model when compared to the human CW. We found the average number of COG IDs per sample was 3860, like the human CW samples (3461) (Fig. 2A), however the overall AS_2_ was 77.98% (Fig. 2C), significantly different and outside the variation observed in human CW (*P* < .0001, Mann–Whitney test) (Fig. 2B).

Looking at the broader functional categories, we found COG IDs related to “virulence” were the least accurate (AS_2_ 64.10%, Fig. S3 and S4A) in the 4-member polymicrobial model. Further, “metabolism” and “cellular processes and signaling” were also poorly captured with AS_2_ of 86.65% and 89.87%, respectively (Fig. S3 and S4B and C). While the overall accuracy score of the 4-member model is significantly lower than the benchmark, this data demonstrates the quantitative framework can be adapted to polymicrobial contexts and reveals functional categories that can be targeted to improve model system accuracy.

### A seven member community captures taxonomic diversity in human CW

**Figure 3:**
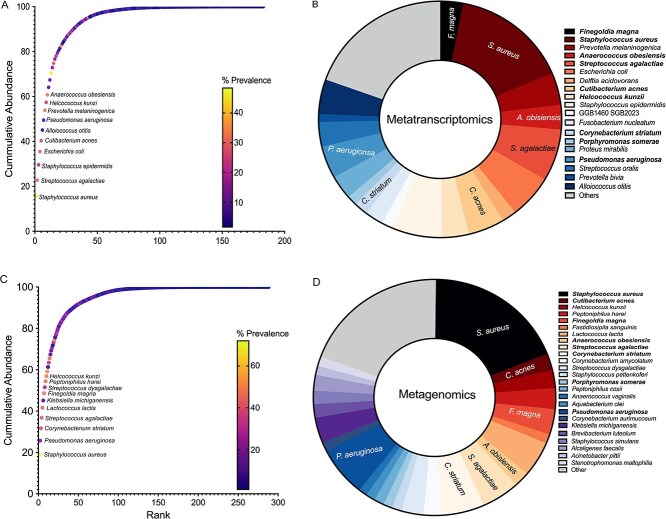
Only a few species comprise the key species in the metatranscriptomic and metagenomic datasets. Graphs show the cumulative abundance of bacterial species in the publicly available data that were evaluated. (A and B) Metatranscriptomic data showing (A) all the species ranked by abundance and colored by prevalence with approximately only top 60% labeled and (B) all species colored from warm to cool by prevalence and size of wedge is abundance highlighting top 80% with distinct colors and other species grouped as “other” colored in grey. (C and D) Metagenomic data showing (C) all the species ranked by abundance and colored by prevalence with approximately only top 60% labeled and (D) all species colored from warm to cool by prevalence and size of wedge is abundance highlighting top 80% with distinct colors and other species grouped as “other” colored in grey.

Next, we considered approaches to rationally improve the accuracy of this experimental model and proposed two key axes we could manipulate: community composition and host environment (Fig. 2D). We first aimed to modify the community composition and build a representative synthetic community. To determine the taxonomic composition of our target, we reanalyzed the 72 human CW meta-transcriptomes with MetaPhlAn4 [39]. Of these 72 samples, MetaPhlAn4 could determine the community composition of 58, identifying a total of 183 species. However, only 141 species had a relative abundance greater than 0.1% in a least 1 sample. We then calculated the cumulative abundance for each species, i.e. considering the abundance for all species across all samples as 100%, how much did each species contribute. We found 46 bacterial species made up over 95% of the cumulative abundance across all samples (Fig. 3A, Dataset S2A), and only 19 bacterial species comprised over 80% of the data (Fig. 3B, Dataset S2A). These highly abundant and prevalent species were a mix of canonical pathogens such as *S. aureus, Streptococcus agalactiae, Staphylococcus epidermidis, Escherichia coli*, and *P. aeruginosa,* and commensal anaerobes such as *Cutibaterium acnes, Anaerococcus obesiensis*, and *F. magna*. Further analysis revealed that *S. aureus* was the most abundant with *F. magna* the most prevalent (Fig. 3A and B).

To further understand CW community composition, we identified 94 human CW metagenomic samples (Table S4) from previously published studies [40, 41]. Using the approach above, we identified 288 total species, however like the metatranscriptomic data, 68 species comprised 95% of the total abundance (Fig. 3C, Dataset S2B) again demonstrating the presence of many bacterial taxa with low abundance and prevalence. Further, we found 25 species contributed to over 80% of the cumulative abundance (Fig. 3D, Dataset S2B). These top species were also a mix of canonical pathogens, such as *S. aureus, S. agalactiae, E. coli, P. aeruginosa,* and commensals such as *Corynebacterium amycolatum, C. acnes, A. obesiensis*, and *F. magna. S. aureus* was the most abundant and the most prevalent (Fig. 3C and D). Interestingly, there was a high degree of overlap between the metagenomic and metatranscriptomic datasets, with nine species highly abundant and prevalent in both datasets, despite no sample or clinical overlaps (Fig. 3B and D, Dataset S2C).

Building off these data, we seeded a 7-member mock community consisting of *S. aureus, C. acnes, F. magna, A. hydrogenalis, S. agalactiae, C. amycolatum, and P. aeruginosa* in WLM*,* as publicly available representatives of genera which were highly abundant and prevalent in both datasets. We targeted a seven-member community as multiple studies have found chronic wounds to contain ~6–7 species per wound with diverse methodology and representatives of these genera have been consistently highlighted as important in CWs [6–9]. Additionally, we found the taxonomic diversity in our synthetic community was represented in some of our human samples (Fig. S5A and B).

We obtained transcriptomic data from the 7-member WLM culture at 48 h post inoculation and found the number of expressed COG IDs was higher than the 4-member community (Fig. 2A). However, the overall AS_2_ of the 7-member community was 78.00% (Fig. 2C) and was not significantly different from the 4-member model (Kruskal-Wallis and Dunn’s multiple comparison tests, *P* > .9999) (Fig. S4F), demonstrating that while our 7-member community better captured the taxonomic diversity and number of expressed COG IDs compared to the human CW, it did not accurately replicate microbial community function. Specifically COG IDs in the categories “virulence,” “metabolism,” and “cellular processes” were poorly captured with AS_2_ scores of 69.23%, 87.7% and 89.97%, respectively (Fig. S3 and Figs S4A–E).

### Microbe-microbe interactions impact the stability of community members and overall community function

Microorganisms in infection environments exhibit several interactions that impact disease progression and overall community physiology [12, 14, 21, 23, 33, 42–46]. Recognizing that interactions between community members may impact the AS_2_, we highlighted two well studied members, *S. aureus* and *P. aeruginosa*, for further investigation. Several studies have shown *P. aeruginosa* exoproducts such as LasA protease, 4-hydroxy-2-heptylquinoline-N-oxide (HQNO) and phenazines support in vitro killing of *S. aureus* [11, 47–49], pp2-. Therefore, we hypothesized we could use the accuracy score framework to evaluate if these known interactions were occurring in situ. We predicted if this antagonistic interaction was occurring in human CW, the AS_2_ of the model would be high when *P. aeruginosa*-*S. aureus* competitive interactions were known to occur in vitro [48, 50]. We noted in the 4- and 7-member communities that *P. aeruginosa* CFU increased overtime, while *S. aureus* CFU were reduced (Fig. S6A). We predicted this was due to killing of *S. aureus* by *P. aeruginosa* in these models, and therefore interventions to increase *S. aureus* fitness would decrease the AS_2_ if this interaction was occurring in human CW and increase the AS_2_ if not. To test this prediction, we modified the inoculation strategy of the 7-member community in WLM to give *S. aureus* a competitive advantage by increasing the ratio of *S. aureus* to *P. aeruginosa*. Importantly, qPCR showed all members of the community were present up to 48 Hours with this inoculation strategy (Fig. S6B). We found this modified inoculum community had an AS_2_ of 82.02% (Fig. 2C and Fig. S3), indicating that lowering the ratio of *P. aeruginosa* compared to other community members increased the accuracy of the experimental model. More importantly, COG IDs supporting “metabolism” and “cellular processes” were increased, with “virulence” and “information storage and processing” slightly reduced when compared to the initial 7-member community (Fig. S3, Fig. S4 and S7). However, while this modified inoculation strategy increased the overall AS_2_, we found that it was not significantly different from the 4-member community in WLM (Kruskal-Wallis and Dunn’s multiple comparison tests, *P* > .9999) (Fig. S4F). We also found *P. aeruginosa* still grew rapidly and *S. aureus* CFU/ml remained reduced in this model (Fig. S6A). Additionally, this data supports the limitation that qPCR data is not an absolute quantification of live bacterial cells. Subsequent experiments with the 7-member community were performed with this altered inoculation strategy.

### The host environment strongly impacts microbial physiology

While modifying community composition is one way to improve model accuracy, microbes are highly responsive to their environments [21, 51–54]. Therefore, we predicted we could instead improve model accuracy by modifying the growth environment (Fig. 2D). Approximately 97% of the human CW metatranscriptomes comprising our benchmark were from diabetic foot ulcers. Therefore, we predicted hyperglycemic WLM may better reflect the infection environment and improve model accuracy. We also predicted the increased glucose may also increase *S. aureus* fitness, as *S. aureus* has multiple glucose transporters it uses to increase its glucose import from its extracellular environment, increasing *S. aureus* virulence [52, 55–57]. We seeded the altered 7-member mock community in hyperglycemic WLM (4.5 g/L glucose) consistent with diabetic blood glucose levels [58–61]. We found this model had an AS_2_ of 77.62% (Fig. 2C) which was not significantly different from the 4-member community in WLM (Kruskal-Wallis and Dunn’s multiple comparison tests, *P* > .9999) (Fig. S4F). We also found “metabolism,” “virulence,” and “cellular processes” had lower AS_2_ in this 7-member hyperglycemic model compared to non-hyperglycemic model in WLM (Fig. 2C and Fig. S3 and S4). Further, we observed while the addition of glucose maintained *S. aureus* up to 24 h post inoculation, by 48 h *S. aureus* had fallen to ~1 × 10^4^ CFU/ml while *P. aeruginosa* had grown to 6 × 10^9^ CFU/ml (Fig. S6A and S7). Collectively, this data is suggestive of *P. aeruginosa* killing *S. aureus* in WLM, similar to previous observations in vitro [48–50, 62].

To further investigate the impact of the host environment on overall community function we infected a murine surgical chronic wound model of infection [33, 45, 63, 64] with the 4- and altered 7-member synthetic communities. At 4 days post infection we found the mean expressed COG IDs of these synthetic communities were 5696 and 4961, respectively (Fig. 2A), and the AS_2_ were 89.70% and 90.78%, respectively (Fig. 2C and Fig. S3). Further, we found the “virulence,” “metabolism,” and “cellular processes” categories which were poorly captured in the in vitro experimental models had improved AS_2_ (Fig. S3 and S4), highlighting the critical role of the infection environment in shaping microbial physiology, and in turn, driving overall community function. While these polymicrobial murine models had higher overall AS_2_ (Fig. 2C), we noted they were not a significant improvement from the 4-member community in WLM (Kruskal-Wallis and Dunn’s multiple comparison tests, *P* = .1716 and *P* = .1969 respectively) (Fig. S4F). We also compared these models directly to the human CW samples and saw the “virulence” (Mann–Whitney test, *P* = .0006 and *P* = .0021 respectively) and “poorly characterized” (Mann–Whitney test, *P* = .0152 and *P* = .0267 respectively) categories were significantly different from the human CW samples, indicating they were not well captured in these models (Figs S4A–E).

### Accuracy of cellular processes and virulence across laboratory models

**Figure 4:**
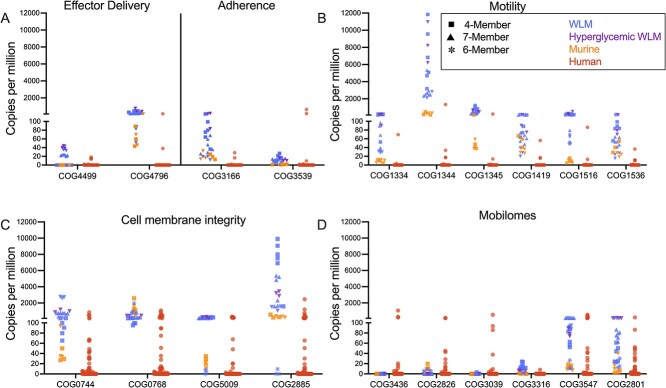
Virulence related functions have higher expression in model samples while genetic mobility is higher in human samples. Graphs show the expression of COG IDs that drive accuracy scores in all the samples evaluated. Y-axis for A–D is the normalized expression (copies per million) for each COG ID. Each point is an individual sample. COD IDs are grouped by subcategories they belong to. Samples are grouped as humans or model. Model samples are shaped by community structure and colored by infection environment.

The accuracy score analysis revealed microbial physiology in CW was not accurately captured in our experimental models across different community compositions and environments (Fig. 2C and S3). Therefore, we sought to understand the role of individual functions on model accuracy. We found the meta-category “virulence” was the least accurate category in the 4-member WLM condition and gradually increased in accuracy across model improvement iterations (Fig. 2C). Within the “virulence” category, the subcategories “effector delivery system”, “adherence,” “cell membrane integrity” and “motility” were the least accurate. For the “effector delivery system” subcategory, COG IDs involved in the Type II, Type VI (T6SS) and Type VII (T7SS) secretion systems (COG4796, COG3157 and COG4499) had significantly higher expression in the in vitro models compared to the murine and human samples (Mann–Whitney test, *P* < .00001) (Fig. 4A). Further, we found expression of these COG IDs in the murine models to be more like the human CW samples. Similarly, COG IDs involved in “adherence” and “motility” (pili: COG3166, COG3539; and flagella: COG1334, COG1344, COG1345, COG1419, COG1516, and COG1536) had significantly higher expression in the experimental models (Mann–Whitney test, *P* < .00001), particularly the in vitro models, compared to the human CW (Fig. 4B). Functions involved in “cell membrane integrity” (COG0744, COG0768, COG5009 and COG2885) also displayed significantly higher expression in vitro compared to our human CW samples (Mann–Whitney test, *P* < .00001) (Fig. 4C). In contrast, the “mobilome” subcategory within the “cellular processes” category was also inaccurate and displayed higher expression in the human CW samples compared to the laboratory models (Fig. 4D). Particularly, multiple transposases (COG2826, COG3436, and COG3039) was expressed significantly higher (Mann–Whitney test *P* < .01) higher in the human samples compared to our experimental models (Fig. 4D). Of note, many inaccurate functions are COG IDs encoded solely by *P. aeruginosa* in our synthetic communities, indicating that inclusion of *P. aeruginosa* in these communities could be driving the reduced accuracy (Fig. S7).

### A six-member polymicrobial community better capture in situ microbial physiology

We observed that *P. aeruginosa* both competitively reduced the burden of *S. aureus* and drove key functions towards reduced accuracy, resulting in community expression profiles that were dissimilar from the human wounds (Fig. S5A and Fig. S7). Therefore, we hypothesized the elimination of *P. aeruginosa* in the 7-member community model would increase model accuracy. We inoculated WLM with a 6-member community comprised of all members of our described 7-member community, but with *P. aeruginosa* omitted, and calculated the AS_2_ 48 h post inoculation as above. We found while there was a significant decrease in the mean number of COG IDs identified (2511) compared to our previous models (Fig. 2A), the AS_2_ of the 6-member community dramatically improved to 96.27% (Fig. 2C and Fig. S3). To verify this improvement was not driven solely by *S. aureus*, we compared the 6-member community to *S. aureus* alone in WLM at 48 h, which yielded fewer expressed COG IDs (mean 1531; Fig. 2A) and a lower AS_2_ (89.80%; Fig. 2C and Fig. S3). We found that there was no significant difference between the overall AS_2_ of the *S. aureus* mono-culture model and that of the initial 4-member community in WLM (Kruskal-Wallis and Dunn’s multiple comparison tests, *P* = .2254), indicating *S. aureus* alone was insufficient to capture human CW community physiology (Fig. S4F). However, notably the 6-member community in WLM was significantly improved from the initial 4-member WLM community (Kruskal-Wallis and Dunn’s multiple comparison tests, *P* = .0111) (Fig. S4F) and was the only experimental model that was significantly improved. Further, when we compared the broader functional categories “cellular processes,” “information storage and processing”, “metabolism,” “virulence” and “poorly characterized,” in these experimental models to the human CW samples we found the 6-member community was not significantly different from the human CW samples across these categories (Mann–Whitney test *P* = .7652, .7557, .1424, .2216 and .1200 respectively) (Figs S4A–E). Importantly, the overall AS2 and AS2 of individual categories of the 6-member replicates were within the variation observed in the human samples (Fig. S9A).

We next examined what functions led to higher accuracy in the 6-member community. Principal component analysis (PCA) revealed the 6-member community and murine models clustered closer to a subset of human CW samples than the remaining in vitro models (Fig. S8A and B), and expression of the top 50 COG IDs driving clustering was more similar between the 6-member community and human CW samples than between the in vitro models (Fig. S8C). Additionally, “virulence” and “cellular process” categories, which were poorly captured in earlier models, improved to 94.87% and 96.24%, respectively (Fig. 2C and Fig. S4A–E). Notably, the “motility” sub-category in the 6-member community matched the low human CW expression levels, suggesting “motility” functions were expressed primarily by *P. aeruginosa* and CW communities are largely non-motile (Fig. 4B). In contrast, functions involved in “cell membrane integrity” remained poorly captured in all experimental models, but particularly in vitro conditions (Fig. 4C). Finally, “mobilome” functions had reduced expression and accuracy (87.5%) relative to both human CW samples and murine models (Fig. 4D).

Finally, we evaluated how *S. aureus* metabolism differed in mono-culture compared to in the 6-member community and the impact on accuracy scores. We saw that 161 metabolism-associated COGs had high expression in *S. aureus* mono-culture, outside the range observed in the human CW, but shifted into the AS_2_ range (Z-score − 2 to +2) in the six-member community (Fig. S9B and C, Dataset S3A and B). These COGs include central carbon metabolism and energy-generation functions such as glycolysis, pentose phosphate pathway, TCA cycle and ATP synthesis (Datasets S3A–C). We further analyzed metabolism-associated COGs whose expression were in the AS_2_ range (Z-score − 2 to +2) in both *S. aureus* mono-culture and the 6-member community (Fig. S9D and E, Datasets S3D–F). We found that in contrast to *S. aureus* mono-culture, multiple taxa contributed to distinct aspects of these conserved COGs (Fig. S9F). For example, *C. amycolatum* drove the expression of amino acid and cofactor biosynthesis, iron acquisition and siderophore-associated functions, and sugar uptake (Dataset S3D–F) while *F. magna* drove the expression of peptide transport and ferredoxin, consistent with anaerobic metabolism and peptide utilization (Dataset S3D–F). Notably, *S. aureus* shifted toward amino acid uptake, nucleotide salvage, and oxidative stress management, consistent with reduced reliance on glycolysis (Dataset S3D–F) in the community compared to mono-culture. *S. agalactiae* maintained high expression of glycolytic and fermentative energy pathways, suggesting it compensates for central carbon flux within the community (Datasets S3D–F).

## Discussion

Laboratory models remain essential tools, particularly in pathogenesis where direct studies are constrained by ethical limitations. Therefore, it is critical to understand the accuracy of experimental models in capturing microbial physiology within the native environment. Recent studies [1, 4, 5] have reported the accuracy of some models, however, none have been done in a polymicrobial context. Here, we provide the first quantitative evaluation of the accuracy of polymicrobial infection model systems, using human CW as a test case. With this extended framework, we can now evaluate the accuracy of polymicrobial models in any biological system using any benchmarked omics data and importantly can use this extended framework to develop more representative polymicrobial models. We used a well-characterized 4-member polymicrobial model as a baseline for iterative experimental model improvement along two axes: community composition and host environment. We built a representative synthetic community, leveraging publicly available metatranscriptomic [26–28] and metagenomic [40, 41] datasets from human chronic wound and our framework to improve the accuracy of this system. Further, we used a range of culture and in vivo conditions to better understand the impact of the host environment on microbial community function and evaluated the expression of these functions in comparison to that in human infection (Fig. S10).

An interesting trend in our data was that “virulence,” and “cellular processes” were the least accurate categories across all model types, and these gradually improved across iterations (Fig. 2C and Fig. S3). We focused on the least accurate sub-categories within “virulence” and “cellular processes” for further exploration into what was driving this inaccuracy. Within the “effector delivery system” sub-category, COG IDs involved in the Type II (T2SS), Type VI (T6SS) and Type VII (T7SS) secretion systems contributed to this reduced accuracy and are known to be involved in bacterial competition [21, 65–69]. The high expression of the T6SS and the T7SS in the in vitro polymicrobial models in this subcategory (Fig. 2C and Fig. 4A) could indicate an over-stimulation of bacterial competition in the models compared to the human CW environment. Interestingly, this subcategory had improved accuracy in the murine models and in the 6-member in vitro model, suggesting *P. aeruginosa* is contributing to that sub-category in vitro and that expression of these systems is reduced in vivo.

Similarly, the “adhesion” and “motility” sub-categories had significantly higher expression of COG IDs related to the bacterial Type VI Pilus and flagellar systems in vitro compared to the human wound samples (Fig. 2C and Fig. 4A and B). *P. aeruginosa*, the sole member of our synthetic communities that encodes for these functions, uses its Type IV Pilus and flagellar systems for motility and to sense surfaces for possible attachment [70, 71]. The high expression of these functions in vitro but not the murine samples indicate that while *P. aeruginosa* possesses this capacity, these structures are likely not expressed at high levels in vivo in wounds. The *Pseudomonas* pilin and the flagellar system have been shown to activate immune responses [72–75]. The high expression of these systems in vitro but not in vivo could suggest the cells are highly motile in vitro and that *P. aeruginosa* reduces expression of these systems in vivo, possibly to avoid host immune activation.

Interestingly, we observed multiple COG IDs involved in “cell membrane integrity” had significantly higher expression in our experimental models compared to the human CW samples (Fig. 4C). The high expression of these membrane integrity proteins in our in vitro and murine laboratory models could be reflective of the stressful environment the cells are in. Membrane integrity related functions often participate in key processes such stress response or cell homeostasis [76, 77]. In vitro the cells are in batch culture, allowing for the accumulation of metabolic waste products and pH shifts, and we found evidence of competitive interactions in these models, further increasing bacterial stress. In addition, the mice have fully active immune systems the microbes need to overcome. This contrasts with human CW infections, which are known to have impaired immune responses [78–84] and our data indicates lower expression of functions associated with microbe-microbe interactions. Therefore, the CW environment may actually induce lower general bacterial stress responses relative to our experimental models.

While multiple inaccurate virulence subcategories had increased expression in the experimental models compared to human CW infection, the sub-category “mobilomes” had significantly higher expression in the human CW samples compared to the laboratory models, driven largely by transposase COG IDs (Fig. 4D). Higher transposase expression in vivo may reflect dynamic selective pressures in the wound infection environment which may require increased genomic flexibility to survive against the sustained stressors of host immunity, nutrient limitation, and polymicrobial competition.

One interesting application of our polymicrobial accuracy score framework is to evaluate if known microbe-microbe interactions occur in human infection by their impact on model accuracy. Several studies have shown antagonistic interactions between *P. aeruginosa* and *S. aureus* in vitro through the production of *P. aeruginosa* exoproducts [11, 47–49, 62]. In support, we found increases in *P. aeruginosa* burdens in our in vitro models were accompanied by decreased *S. aureus*, and the models with this trend had lower accuracy (Fig. 2C, Figs S4, S6A, and  S7). Further, *P. aeruginosa* dominated the expression of key categories in these models (Fig. S7). In contrast, the murine models where previous work has shown co-existence of *S. aureus* and *P. aeruginosa* [45] had improved accuracy (Fig. 2C and Fig. S4), and had increased *S. aureus* expression of key categories (Fig. S7). The observation that conditions with reduced *S. aureus* and higher abundance of *P. aeruginosa* were less accurate suggested that a competitive interaction was occurring in vitro and that this antagonism is not a key driver of microbial community function in human CW infections. Supportive of this idea, omission of *P. aeruginosa* in the 6-member community was accompanied by the increased *S. aureus* bacterial burdens (Fig. S6A), increased expression of key categories by *S. aureus* and other community members, and an increased accuracy score that matched the variation within the human CW samples (Fig. 2C and Fig. S9A). Importantly, the 6-member WLM condition had improved coverage of functions that were poorly captured in the previous models (Figs 4A–D and Figs S4A–E) further demonstrating that reducing the expression of those functions, here by omission, was key to mimicking in situ microbial physiology. Further, the community contribution to functions in the 6-member community better represents the pattern observed in our human CW samples (Fig. S5A and Fig. S7). While it is unlikely that human chronic wounds lack *P. aeruginosa*, this data suggests that the competitive advantage of *P. aeruginosa* in vitro does not mimic the human CW infection environment. Future studies will seek to better understand the molecular mechanisms driving model accuracy. Together, these will increase our understanding of the microbial community functions in human CW infections and provide insights into the microbe-microbe interactions that occur in situ.

While we believe our polymicrobial accuracy score approach is a powerful tool for understanding the complex biological processes and interactions within polymicrobial communities, there are some key limitations. We selected COG classification in our approach; however, this choice has trade-offs. GO and KO terms offer detailed functional information but are biased towards well-annotated organisms, and for GO terms functions are collapsed into only three broad categories with frequent overlaps, making the interpretation challenging. Level4EC focuses on enzymes with known catalytic activity, limiting its utility for uncharacterized proteins. PFAM enables domain-based predictions but showed limited coverage as few UniRef90 IDs had direct PFAM IDs conversions. In contrast, COG classification grouped orthologs across taxa to into hierarchical functional for further traceability and extensive analysis. However, a key limitation is COG databases are not always up to date, leaving many IDs poorly characterized. These challenges highlight the need for improved annotation resources as accurate annotation is critical for translating omics data into biological insights. Further, our 7-member synthetic community included publicly available bacterial isolates representative of the most abundant and prevalent genera in human CWs. However, due to isolate availability, we substituted *Anaerococcus obesiensis* with a closely related publicly available species, *A. hydrogenalis*, and substituted *Corynebacterium striatum* with a publicly available *C. amycolatum* isolate in our synthetic community. While both species were also found in our datasets, choices in which isolates and strains to include is a key limitation. Additionally, the use of transcriptomics may be biased towards a single organism where highly expressed genes dominate the dataset.

In conclusion, we demonstrated the application of an extended framework to develop accurate polymicrobial models in any system. Further, we propose a 6-member synthetic community that captures the taxonomic diversity and functional complexity of human chronic wound infections that can be applied to future work. Importantly, our framework can be used to evaluate whether known in vitro microbe-microbe interactions occur in situ and this can be applied to the evaluation of any environment of interest using any benchmarked omics data. Collectively, this approach is an important next step towards accurate model systems that reflect the biological processes they are meant to capture.

## Supplementary Material

ycag061_Supplementary_material

## Data Availability

Codes for the analyses in this manuscript can be found on our GitHub page (https://github.com/Ibberson-Lab/Assessing-Microbial-Community-Physiology-in-a-Polymicrobial-Infection). The raw sequencing files for generated RNA-seq data are submitted to SRA under the accession number PRJNA1314253.
