# Supplementary material for: Revealing community dynamics in polymicrobial infections through a quantitative framework
Source: ISME Commun. 2026 Mar 13;6(1):ycag061. doi: 10.1093/ismeco/ycag061 (PMC13064647; doi:10.1093/ismeco/ycag061)
Supplement: ycag061_Supplementary_material [file ycag061_supplementary_material.zip › Supplementary_material_ycag061/SuppInfo_FiguresTables_ISMEComms_resub_final_accepted.docx]

**Supplementary Information for**

**Revealing Community Dynamics in Polymicrobial Infections through a Quantitative Framework**

Aanuoluwa E. Adekoya,^1^ Tyler E. Boggs,^1^ and Carolyn B. Ibberson^1^

^1^Department of Microbiology, University of Tennessee, Knoxville, TN, USA

Correspondence may be addressed to Carolyn B. Ibberson.

Email: [ibberson@utk.edu](mailto:ibberson@utk.edu)

This PDF file includes:

Materials and Methods

Figures S1 to S10

Tables S1 to S4

Legends for Datasets S1 to S3

SI References

**Materials and Methods**

**Bacterial Strains for Model and Growth Conditions**

Bacterial strains used in this study are listed in Table S1. For the four-member polymicrobial model *Staphylococcus aureus*, *Pseudomonas aeruginosa*, *Finegoldia magna* and *E. faecalis* were used as previously described [1]. The following bacteria were incorporated in the seven-member community: *Staphylococcus aureus*, *Pseudomonas aeruginosa*, *Streptococcus agalactiae*, *Cutibacterium acnes*, *Finegoldia magna*, *Anaerococcus hydrogenalis*, and *Corynebacterium amycolatum*. Culture conditions are detailed in the supplementary material. To prep bacterial inoculum for the experimental models, *S. aureus*, *P. aeruginosa* and *E. faecalis* grown overnight at 37^o^C in Brain Heart Infusion broth (BHI) with shaking at 150 RPM (Innova 44 Incubator Shaker series). *C. amycolatum* was grown in BHI + 1% Tween in the same condition. *S. agalactiae* was grown statically at 37^o^C in Todd Hewitt Broth (THB). *F. magna* and *A. hydrogenalis* were grown at 37^o^C in MTGE anaerobic enrichment broth under anaerobic conditions (Anaerobes Systems AS-150 with 5% Hydrogen and Nitrogen balance) with *C. acnes* being grown for 4 days.

For the default four-member and seven-member polymicrobial model, equal volume of each species was used to generate an inoculum at approximately 1 X 10^6^ colony forming units (CFU)/ml. For the 7-member altered inoculum, *P. aeruginosa* was normalized to an OD_600_ of 0.01, *S. aureus* and *S. agalactiae* were normalized to an OD_600_ of 0.02, and *F. magna*, *C. acnes*, *C. amycolatum* and *A. hydrogenalis* were normalized to an OD_600_ of 0.1. Then, equal volume of each species was used to make the inoculum and this community (60 μl was inoculated into 540 μl of Lubbock Wound-Like Media [2] in 96-well deep-well plates (Eppendorf 96/2000 uL) and grown statically at 37^o^C in ambient air for 48 hours. At 48 hours, samples were collected and stored in Zymo DNA/RNA shield (Zymo Research R1100-250) for DNA and RNA extraction. Two technical replicates were combined for each biological replicate.

**Oxygen Concentration**

Using the Unisense microprofiling system with an oxygen microsensor 25µM probe (OX-25), we measured oxygen concentration in four replicates of each model culture over time (0 h, 24 h and 48 h). Our culture surface was 11mm from the bottom, then we measured oxygen concentration (µmol/L) at the top (~1mm from surface), middle (~4mm from surface) and bottom (~7mm from surface) for each time point.

**In-Vivo Model of Chronic Wound Infection**

8- to 10-weeks-old female C57BL/6J mice (Jackson’s Lab) were used to perform the murine surgical wound infections as previously described [3–6]. Briefly, the OD_600_ of the initially described species were reduced to 0.05 to obtain approximately 5 × 10^5^ CFU in the inoculum for each condition (n = 4). Mice were anesthetized using isoflurane, their backs were shaved and administered bupivacaine. A full thickness 1.5 X 1.5 cm surgical excisional wounds were generated on the dorsal skin, covered with a transparent semi permeable polyurethan dressing. The inoculum for each condition was injected directly under the dressing. At 4 days post infection (4dpi), mice were euthanized, and the wound tissues were excised and immediately saved in 750ul of Zymo DNA/RNA shield (Zymo Research R1100-250) for preservation for DNA and RNA extraction. The protocol for this study was approved by Institutional Animal Care and Use Committee of the University of Tennessee Knoxville (Protocol Number 3071), and all animals were cared for and handled according to the recommendations provided.

**Quantitative PCR (qPCR) Analysis to Determine Bacterial Burden**

qPCR analysis was performed to determine bacterial burden at time of sample collection. Briefly, genomic DNA was extracted from invitro and murine samples using the ZymoBIOMICS DNA/RNA Miniprep Kit (R2002) and quantified using Qubit RNA broad range assay (ThermoFisher Q10210) and RNA IQ assay (ThermoFischer Q33221) kits on the Qubit fluorometer (ThermoFisher Q33238). Genomic DNA was also extracted from overnight standards for standard curve quantification. The DNA for each standard curve was diluted 1:10 fold for five dilutions. The universal 16S primers and species-specific primers (Table S2) were used for all bacteria expect for *A. hydrogenalis.* As some of these conditions had numerous bacterial species, end-point PCR assays were performed to test the specificity and cross-reactivity of each primer. Power SYBR (Applied Biosystems Power SYBR™ Green PCR Master Mix) in 20 μl reactions were used for qPCR on Applied Biosystems QuantStudio™ 3 Real-Time PCR System (ThermoFisher) with the following steps 95°C for 10 minutes, and 40 cycles of 95°C for 15 seconds and 60°C for 60 seconds. Unknown bacterial quantification was done in comparison to the standard curve.

**RNA-sequencing**

In vitro and murine wound tissues were collected in DNA/RNA shield as described above. RNA extraction was performed using ZymoBIOMICS DNA/RNA Miniprep Kit (R2002) and the Qiagen RNeasy Mini Kit (Qiagen 73404) with Qiazol (Qiagen 79306). Our preliminary experiments with head-to-head comparisons of these two RNA extraction kits from the same samples we did not observe significant impacts to the resulting sequencing data, as evaluated by clustering and differential expression analyses. The extracted RNA was prepared for sequencing using an Illumina Stranded Total RNA Prep Ligation Kit with Ribo-Zero Plus Microbiome (#20072063) with Illumina Unique Dual Indexes by SeqCoast Genomics. Sequencing was performed on the Illumina NextSeq2000 platform using a 300-cycle flow cell kit to produce 2x150bp paired reads. 1-2% PhiX control was spiked into the run to support optimal base calling. At least 3 biological replicates of each condition in were used.

**Human Data Collection**: We collected 72 metatranscriptomic datasets [7–9] identified as being from chronic wounds fom the Sequence Reads Archive (SRA) (Table S3). These sequencing files were originally submitted with the project IDs: PRJNA563930, PRJNA726011, PRJNA720438, and SRP135669. Only SRP135669 was not a diabetic ulcer project. To enrich our community composition data, we further analyzed 95 metagenomic datasets. The metagenomic datasets were from PRJNA506988, which had 195 samples (100 of the 195 samples were non-duplicates) and PRJNA610303 (36 samples); however, only 94 were individual samples from chronic wounds [10,11]. Since the PRJNA506988 study was a longitudinal study, chronicity was determined by a third visit to the facility, which was after 26 weeks of follow-up. All of these were diabetic foot ulcers/infections. All metagenomic samples used are listed in Table S4.

**Community Composition and Functional Analysis**

Using only the forward reads for metatranscriptomic data and paired reads for the metagenomics data, FastQC v0.11.5 [12] was used for quality check, and CutAdapt [13] was used for adapter sequence removal and trimming. SPAdes genome assembler v4.0.0 [14] was used for genome assembly for the metagenome data. Cutadapt v5.0 [13] with universal Illumina adapter AGATCGGAAGAGCACACGTCTGAACTCCAGTCAC was used to remove the adapter sequences and reads with less than 22 bases in length. For the human chronic wounds sample, the trimmed reads (cutadapt output) were mapped to the human genome reference (GRCh38/hg38) using bowtie v2.5.4 [15]. The prokaryotic reads were run through SortMeRNA v4.3.7 [16] was used to remove the ribosomal rRNA. (Fig. S1A). For community composition, MetaPhlAn v4.1 [17], using the Oct2024 SBG CHOCOPhlAn database (CHOCOPhlAnSGB), was used to bin all the prokaryotic reads from the human CW and the in vitro samples to microbes or obtain the community composition data. (Fig. S1A). For community function analysis**,** HUMANn v4.1.1 alpha release [18] with taxonomic profiling from MetaPhlAn v4.1 [17] and other default parameters was used to obtain the metabolic potential data of all the samples in our dataset. The output of these was in UniRef90 IDs and was already normalized for sequencing depth differences to Copies Per Million reads (CPM). We further regrouped the UniRefIDs to EggNOG categories (COG (Clusters of Orthologous Genes) IDs) using the humann_regroup_table function in HUMANn v4.1.1 alpha. The functional categories of these COG IDs were obtained from the eggnog5.0 database FTP (<http://eggnog5.embl.de/download/eggnog_5.0/>), the COG API linkage on NCBI <https://www.ncbi.nlm.nih.gov/research/cog/api/cogdef/>, the KOG database on NCBI <https://ftp.ncbi.nlm.nih.gov/pub/COG/KOG/kog> and the species annotation from MaGe [19].

**Accuracy Score Analysis**: To reduce noise in the datasets, COG IDs whose cumulative fraction did not contribute to up to 99% of the entire COG ID expression were removed from subsequent analyses. The quantitative framework was used as previously described [20–22] to determine the accuracy of each COG ID and the overall model, using expression in the human chronic wound samples as the target. In brief, ​the mean expression of each COG ID and the deviation from the human CW samples was obtained as a Z score. The percentage of the COG IDs that fall within 2 standard deviations from the human CW samples was recorded as the accuracy score (AS_2_) for each functional category. We performed leave-two-out cross validation where 2 random human CW samples were selected and compared to the remaining human CW samples. This step was repeated 1200 times, and the average AS_2_ score was calculated based on the number of iterations and was used as the baseline AS_2_ for comparison. All statistical analyses were done using RStudio (R v4.4.1) and GraphPad Prism v11.0.0. Some figures were generated using Biorender.

**Table S1.** Strains used in this study.

| Strain | Description | Identifier | Source or Reference |
| --- | --- | --- | --- |
| LAC | *S. aureus* LAC* (AH1263) community associated methicillin resistant USA300 isolate | CI3 | [23] |
| PAO1 | *P. aeruginosa* strain PAO1 wildtype strain | CI91 | [24] |
| SY01 | *F. magna* strain SY01 (HM-293), isolated from the vagina of a patient with bacterial vaginosis in Urbana, Illinois. Obtained from BEI Resources as part of the Human Microbiome Project. | CI201 | BEI Resources |
| V583 | *E. faecalis* vancomycin resistant isolate V583 | CI164 | [25] |
| A909 | *S. agalactiae* strain A909 isolated in 1934 from a septic human neonate. | CI185 | [26] |
| SK137 | *Cutibacterium acnes* (formerly *Propionibacterium acnes*) isolate SK137 (HM-122), isolated from the normal skin of a 57-year-old man. Obtained from BEI Resources as part of the Human Microbiome Project. | CI232 | BEI Resources |
| MJR7738A | *A. hydrogenalis* MJR7738A | CI233 | BEI Resources |
| SK46 | *C. amycolatum* strain SK46 (HM- 109D), isolated from the normal skin of a 57-year-old man. Obtained from BEI Resources as part of the Human Microbiome Project. | CI199 | BEI Resources |

**Table S2.** Quantitative PCR primers used in this study. All sequences are listed 5’ to 3’.

| *Species* | Forward | Reverse | Source |
| --- | --- | --- | --- |
| *P. aeruginosa* | TAAGGACAGCCAGGACTACGAGAA | TGGTAGATGGACGGTTCCCAGAAA | [27] |
| *S. aureus* | ATTTGGTCCCAGTGGTGTGGGTAT | GCTGTGACAATTGCCGTTTGTCGT | [27] |
| *F. magna* | TACTAATGAGAGTGGCGAACGGGT | ATTAATCCCGGTTTCCCGAGGCTA | [27] |
| *E. faecalis* | ACCAAGCGGCGTCAAGTATCAAGA | GTGTGCGCAATCGCTCCAATTTCT | [27] |
| All bacteria (Universal 16S) | CCATGAAGTCGGAATCGCTAG | GCTTGACGGGCGGTGT | [27] |
| *C. acnes* | GACATGGATCGGGAGTGCTC | CATAACGTGCTGGCAACAGTG | [28] |
| *S. agalactiae* | CACTCAGCTTGGGATAGATCAG | GAAGAGTCAGGTTCGGTCATT | This study |
| *C. amycolatum* | GGTTGATGTTGCGCTTTACC | GGCAGACTCTCGGAAAGAAA | This study |

**Table S3.** Metatranscriptomic samples used in this analysis. Red text indicates samples that were omitted from subsequent analyses following quality control. DFU = Diabetic Foot Ulcer

| **SRR ID** | **Source (Country)** | **City/State** | **Patient Age** | **Patient Sex** | **Infection type** | **Accession Number** |
| --- | --- | --- | --- | --- | --- | --- |
| SRR10074438 | Australia | Liverpool |  |  | DFU | PRJNA563930 |
| SRR10074439 | Australia | Liverpool | >18 | M | DFU | PRJNA563930 |
| SRR10074440 | Australia | Liverpool | 61 | F | DFU | PRJNA563930 |
| SRR10074441 | Australia | Liverpool | 68 | M | DFU | PRJNA563930 |
| SRR10074442 | Australia | Liverpool | 62 | M | DFU | PRJNA563930 |
| SRR10074443 | Australia | Liverpool |  |  | DFU | PRJNA563930 |
| SRR10074444 | Australia | Liverpool | 77 | M | DFU | PRJNA563930 |
| SRR10074445 | Australia | Liverpool | 71 | M | DFU | PRJNA563930 |
| SRR10074446 | Australia | Liverpool |  |  | DFU | PRJNA563930 |
| SRR10074447 | Australia | Liverpool |  |  | DFU | PRJNA563930 |
| SRR10074448 | Australia | Liverpool | 72 | M | DFU | PRJNA563930 |
| SRR10074449 | Australia | Liverpool |  |  | DFU | PRJNA563930 |
| SRR10074450 | Australia | Liverpool | 51 | F | DFU | PRJNA563930 |
| SRR10074451 | Australia | Liverpool | 68 | M | DFU | PRJNA563930 |
| SRR10074452 | Australia | Liverpool | 51 | F | DFU | PRJNA563930 |
| SRR10074453 | Australia | Liverpool | 64 | M | DFU | PRJNA563930 |
| SRR14174610 | Australia | Sydney | 60 | M | DFU | PRJNA720438 |
| SRR14174611 | Australia | Sydney | 54 | M | DFU | PRJNA720438 |
| SRR14174612 | Australia | Sydney |  |  | DFU | PRJNA720438 |
| SRR14174613 | Australia | Sydney | 54 | M | DFU | PRJNA720438 |
| SRR14174614 | Australia | Sydney |  |  | DFU | PRJNA720438 |
| SRR14174615 | Australia | Sydney |  |  | DFU | PRJNA720438 |
| SRR14174616 | Australia | Sydney | 69 | M | DFU | PRJNA720438 |
| SRR14174617 | Australia | Sydney | 38 | M | DFU | PRJNA720438 |
| SRR14174618 | Australia | Sydney |  |  | DFU | PRJNA720438 |
| SRR14174619 | Australia | Sydney |  |  | DFU | PRJNA720438 |
| SRR14174620 | Australia | Sydney |  |  | DFU | PRJNA720438 |
| SRR14174621 | Australia | Sydney | 54 | M | DFU | PRJNA720438 |
| SRR14174622 | Australia | Sydney | 54 | M | DFU | PRJNA720438 |
| SRR14174623 | Australia | Sydney | 51 | M | DFU | PRJNA720438 |
| SRR14174624 | Australia | Sydney | 51 | M | DFU | PRJNA720438 |
| SRR14174625 | Australia | Sydney | 51 | M | DFU | PRJNA720438 |
| SRR14174626 | Australia | Sydney |  |  | DFU | PRJNA720438 |
| SRR14174627 | Australia | Sydney |  |  | DFU | PRJNA720438 |
| SRR14174628 | Australia | Sydney |  |  | DFU | PRJNA720438 |
| SRR14174629 | Australia | Sydney | 38 | M | DFU | PRJNA720438 |
| SRR14374244 | Australia | Liverpool | 62 | M | DFU | PRJNA726011 |
| SRR14374245 | Australia | Liverpool | 68 | F | DFU | PRJNA726011 |
| SRR14374246 | Australia | Liverpool | 57 | M | DFU | PRJNA726011 |
| SRR14374247 | Australia | Liverpool | 56 | M | DFU | PRJNA726011 |
| SRR14374248 | Australia | Liverpool | 49 | M | DFU | PRJNA726011 |
| SRR14374249 | Australia | Liverpool | 46 | M | DFU | PRJNA726011 |
| SRR14374257 | Australia | Liverpool | 70 | M | DFU | PRJNA726011 |
| SRR14374258 | Australia | Liverpool | 42 | M | DFU | PRJNA726011 |
| SRR14374259 | Australia | Liverpool | 37 | M | DFU | PRJNA726011 |
| SRR14374260 | Australia | Liverpool |  |  | Unknown | PRJNA726011 |
| SRR14374261 | Australia | Liverpool | 36 | F | DFU | PRJNA726011 |
| SRR14374262 | Australia | Liverpool | 34 | F | DFU | PRJNA726011 |
| SRR14374263 | Australia | Liverpool | 34 | F | DFU | PRJNA726011 |
| SRR14374264 | Australia | Liverpool | 60 | M | DFU | PRJNA726011 |
| SRR14374265 | Australia | Liverpool | 69 | M | DFU | PRJNA726011 |
| SRR14374266 | Australia | Liverpool | 64 | M | DFU | PRJNA726011 |
| SRR14374267 | Australia | Liverpool | 46 | F | DFU | PRJNA726011 |
| SRR14374268 | Australia | Liverpool | 45 | M | DFU | PRJNA726011 |
| SRR14374269 | Australia | Liverpool | 64 | M | DFU | PRJNA726011 |
| SRR14374270 | Australia | Liverpool | 68 | M | DFU | PRJNA726011 |
| SRR14374271 | Australia | Liverpool | 51 | M | DFU | PRJNA726011 |
| SRR14374272 | Australia | Liverpool | 59 | M | DFU | PRJNA726011 |
| SRR14374273 | Australia | Liverpool | 56 | M | DFU | PRJNA726011 |
| SRR14374274 | Australia | Liverpool | 71 | M | DFU | PRJNA726011 |
| SRR14374275 | Australia | Liverpool | 71 | M | DFU | PRJNA726011 |
| SRR14374276 | Australia | Liverpool | 55 | M | DFU | PRJNA726011 |
| SRR14374277 | Australia | Liverpool | 67 | M | DFU | PRJNA726011 |
| SRR14374278 | Australia | Liverpool | 67 | M | DFU | PRJNA726011 |
| SRR14374279 | Australia | Liverpool | 64 | M | DFU | PRJNA726011 |
| SRR14374280 | Australia | Liverpool | 39 | M | DFU | PRJNA726011 |
| SRR6833323 | USA | Lubbock, TX | 36 | M | Foot Ulcer | SRP135669 |
| SRR6833324 | USA | Lubbock, TX | 88 | M | Foot Ulcer | SRP135669 |
| SRR6833325 | USA | Lubbock, TX | 31 | M | Foot Ulcer | SRP135669 |
| SRR6833340 | USA | Lubbock, TX | 59 | F | Foot Ulcer | SRP135669 |
| SRR6833343 | Denmark | Copenhagen | 69 | M | Foot Ulcer | SRP135669 |
| SRR6833348 | Denmark | Copenhagen |  |  | Foot Ulcer | SRP135669 |

**Table S4.** Metagenomic samples used in this analysis. DFU = Diabetic Foot Ulcer

| **SRRID** | **Source (Country)** | **City/State** | **Infection type** | **Accession Number** | **Diabetes Type** | **Age** | **Sex** |
| --- | --- | --- | --- | --- | --- | --- | --- |
| SRR8247667 | United States | Pennylvania | DFU | PRJNA506988 | Type 2 | 56 | Female |
| SRR8247668 | United States | Pennylvania | DFU | PRJNA506988 | Type 2 | 56 | Female |
| SRR8247671 | United States | Pennylvania | DFU | PRJNA506988 | Type 2 | 52 | Female |
| SRR8247672 | United States | Pennylvania | DFU | PRJNA506988 | Type 2 | 52 | Female |
| SRR8247673 | United States | Pennylvania | DFU | PRJNA506988 | Type 2 | 52 | Female |
| SRR8247674 | United States | Pennylvania | DFU | PRJNA506988 | Type 2 | 52 | Female |
| SRR8247675 | United States | Pennylvania | DFU | PRJNA506988 | Type 2 | 52 | Female |
| SRR8247676 | United States | Pennylvania | DFU | PRJNA506988 | Type 2 | 52 | Female |
| SRR8247677 | United States | Pennylvania | DFU | PRJNA506988 | Type 2 | 52 | Female |
| SRR8247678 | United States | Pennylvania | DFU | PRJNA506988 | Type 2 | 52 | Female |
| SRR8247679 | United States | Pennylvania | DFU | PRJNA506988 | Type 2 | 52 | Female |
| SRR8247682 | United States | Pennylvania | DFU | PRJNA506988 | Type 2 | 56 | Female |
| SRR8247683 | United States | Pennylvania | DFU | PRJNA506988 | Type 2 | 56 | Female |
| SRR8247686 | United States | Pennylvania | DFU | PRJNA506988 | Type 2 | 60 | Male |
| SRR8247689 | United States | Pennylvania | DFU | PRJNA506988 | Type 2 | 66 | Male |
| SRR8247690 | United States | Pennylvania | DFU | PRJNA506988 | Type 2 | 66 | Male |
| SRR8247693 | United States | Pennylvania | DFU | PRJNA506988 | Type 2 | 58 | Male |
| SRR8247694 | United States | Pennylvania | DFU | PRJNA506988 | Type 2 | 58 | Male |
| SRR8247695 | United States | Pennylvania | DFU | PRJNA506988 | Type 2 | 58 | Male |
| SRR8247696 | United States | Pennylvania | DFU | PRJNA506988 | Type 2 | 58 | Male |
| SRR8247697 | United States | Pennylvania | DFU | PRJNA506988 | Type 2 | 58 | Male |
| SRR8247698 | United States | Pennylvania | DFU | PRJNA506988 | Type 2 | 58 | Male |
| SRR8247699 | United States | Pennylvania | DFU | PRJNA506988 | Type 2 | 58 | Male |
| SRR8247700 | United States | Pennylvania | DFU | PRJNA506988 | Type 2 | 58 | Male |
| SRR8247701 | United States | Pennylvania | DFU | PRJNA506988 | Type 2 | 58 | Male |
| SRR8247704 | United States | Pennylvania | DFU | PRJNA506988 | Type 1 | 37 | Male |
| SRR8247707 | United States | Pennylvania | DFU | PRJNA506988 | Type 2 | 52 | Male |
| SRR8247708 | United States | Pennylvania | DFU | PRJNA506988 | Type 2 | 52 | Male |
| SRR8247711 | United States | Pennylvania | DFU | PRJNA506988 | Type 2 | 52 | Female |
| SRR8247712 | United States | Pennylvania | DFU | PRJNA506988 | Type 2 | 52 | Female |
| SRR8247713 | United States | Pennylvania | DFU | PRJNA506988 | Type 2 | 52 | Female |
| SRR8247714 | United States | Pennylvania | DFU | PRJNA506988 | Type 2 | 52 | Female |
| SRR8247715 | United States | Pennylvania | DFU | PRJNA506988 | Type 2 | 52 | Female |
| SRR8247716 | United States | Pennylvania | DFU | PRJNA506988 | Type 2 | 52 | Female |
| SRR8247717 | United States | Pennylvania | DFU | PRJNA506988 | Type 2 | 52 | Female |
| SRR8247718 | United States | Pennylvania | DFU | PRJNA506988 | Type 2 | 52 | Female |
| SRR8247721 | United States | Pennylvania | DFU | PRJNA506988 | Type 2 | 58 | Female |
| SRR8247722 | United States | Pennylvania | DFU | PRJNA506988 | Type 2 | 58 | Female |
| SRR8247725 | United States | Pennylvania | DFU | PRJNA506988 | Type 2 | 58 | Male |
| SRR8247728 | United States | Pennylvania | DFU | PRJNA506988 | Type 2 | 48 | Male |
| SRR8247729 | United States | Pennylvania | DFU | PRJNA506988 | Type 2 | 48 | Male |
| SRR8247732 | United States | Pennylvania | DFU | PRJNA506988 | Type 2 | 53 | Male |
| SRR8247733 | United States | Pennylvania | DFU | PRJNA506988 | Type 2 | 53 | Male |
| SRR8247736 | United States | Pennylvania | DFU | PRJNA506988 | Type 2 | 49 | Female |
| SRR8247737 | United States | Pennylvania | DFU | PRJNA506988 | Type 2 | 49 | Female |
| SRR8247738 | United States | Pennylvania | DFU | PRJNA506988 | Type 2 | 49 | Female |
| SRR8247739 | United States | Pennylvania | DFU | PRJNA506988 | Type 2 | 49 | Female |
| SRR8247740 | United States | Pennylvania | DFU | PRJNA506988 | Type 2 | 49 | Female |
| SRR8247741 | United States | Pennylvania | DFU | PRJNA506988 | Type 2 | 49 | Female |
| SRR8247742 | United States | Pennylvania | DFU | PRJNA506988 | Type 2 | 49 | Female |
| SRR8247743 | United States | Pennylvania | DFU | PRJNA506988 | Type 2 | 49 | Female |
| SRR8247748 | United States | Pennylvania | DFU | PRJNA506988 | Type 2 | 55 | Male |
| SRR8247751 | United States | Pennylvania | DFU | PRJNA506988 | Type 2 | 62 | Male |
| SRR8247754 | United States | Pennylvania | DFU | PRJNA506988 | Type 2 | 69 | Male |
| SRR8247759 | United States | Pennylvania | DFU | PRJNA506988 | Type 2 | 47 | Male |
| SRR8247764 | United States | Pennylvania | DFU | PRJNA506988 | Type 2 | 58 | Male |
| SRR8247765 | United States | Pennylvania | DFU | PRJNA506988 | Type 2 | 58 | Male |
| SRR8247766 | United States | Pennylvania | DFU | PRJNA506988 | Type 2 | 58 | Male |
| SRR11248549 | Australia | Liverpool | DFU | PRJNA610303 | Type 1 | 54 | Male |
| SRR11248550 | Australia | Liverpool | DFU | PRJNA610303 | Type 1 | 51 | Female |
| SRR11248551 | Australia | Liverpool | DFU | PRJNA610303 | Type 1 | 59 | Male |
| SRR11248552 | Australia | Liverpool | DFU | PRJNA610303 | Type 2 | 54 | Male |
| SRR11248553 | Australia | Liverpool | DFU | PRJNA610303 | Type 2 | 63 | Male |
| SRR11248554 | Australia | Liverpool | DFU | PRJNA610303 | Type 2 | 50 | Female |
| SRR11248555 | Australia | Liverpool | DFU | PRJNA610303 | Type 2 | 65 | Male |
| SRR11248556 | Australia | Liverpool | DFU | PRJNA610303 | Type 2 | 88 | Female |
| SRR11248557 | Australia | Liverpool | DFU | PRJNA610303 | Type 2 | 77 | Male |
| SRR11248558 | Australia | Liverpool | DFU | PRJNA610303 | Type 2 | 50 | Male |
| SRR11248559 | Australia | Liverpool | DFU | PRJNA610303 | Type 2 | 82 | Female |
| SRR11248560 | Australia | Liverpool | DFU | PRJNA610303 | Type 2 | 62 | Male |
| SRR11248561 | Australia | Liverpool | DFU | PRJNA610303 | Type 2 | 53 | Male |
| SRR11248562 | Australia | Liverpool | DFU | PRJNA610303 | Type 2 | 61 | Female |
| SRR11248563 | Australia | Liverpool | DFU | PRJNA610303 | Type 2 | 71 | Male |
| SRR11248564 | Australia | Liverpool | DFU | PRJNA610303 | Type 2 | 54 | Male |
| SRR11248565 | Australia | Liverpool | DFU | PRJNA610303 | Type 2 | 67 | Female |
| SRR11248566 | Australia | Liverpool | DFU | PRJNA610303 | Type 2 | 61 | Female |
| SRR11248567 | Australia | Liverpool | DFU | PRJNA610303 | Type 1 | 51 | Female |
| SRR11248568 | Australia | Liverpool | DFU | PRJNA610303 | Type 1 | 57 | Male |
| SRR11248569 | Australia | Liverpool | DFU | PRJNA610303 | Type 2 | 51 | Male |
| SRR11248570 | Australia | Liverpool | DFU | PRJNA610303 | Type 2 | 64 | Male |
| SRR11248571 | Australia | Liverpool | DFU | PRJNA610303 | Type 2 | 67 | Male |
| SRR11248572 | Australia | Liverpool | DFU | PRJNA610303 | Type 2 | 72 | Male |
| SRR11248573 | Australia | Liverpool | DFU | PRJNA610303 | Type 2 | 71 | Male |
| SRR11248574 | Australia | Liverpool | DFU | PRJNA610303 | Type 2 | 54 | Male |
| SRR11248575 | Australia | Liverpool | DFU | PRJNA610303 | Type 2 | 67 | Male |
| SRR11248576 | Australia | Liverpool | DFU | PRJNA610303 | Type 2 | 69 | Male |
| SRR11248577 | Australia | Liverpool | DFU | PRJNA610303 | Type 2 | 68 | Male |
| SRR11248578 | Australia | Liverpool | DFU | PRJNA610303 | Type 2 | 54 | Male |
| SRR11248579 | Australia | Liverpool | DFU | PRJNA610303 | Type 2 | 81 | Female |
| SRR11248580 | Australia | Liverpool | DFU | PRJNA610303 | Type 2 | 66 | Male |
| SRR11248581 | Australia | Liverpool | DFU | PRJNA610303 | Type 1 | 48 | Male |
| SRR11248582 | Australia | Liverpool | DFU | PRJNA610303 | Type 1 | 36 | Female |
| SRR11248583 | Australia | Liverpool | DFU | PRJNA610303 | Type 2 | 68 | Male |
| SRR11248584 | Australia | Liverpool | DFU | PRJNA610303 | Type 2 | 64 | Male |

**SI References:**

1. Dalton T, Dowd SE, Wolcott RD *et al.* An In Vivo Polymicrobial Biofilm Wound Infection Model to Study Interspecies Interactions. *PLoS ONE* 2011;**6**(11):e27317. https://doi.org/10.1371/journal.pone.0027317.

2. Sun Y, Dowd SE, Smith E *et al.* In vitro multispecies Lubbock chronic wound biofilm model. *Wound Repair Regen* 2008;**16**(6):805–13. https://doi.org/10.1111/j.1524-475X.2008.00434.x.

3. Ibberson CB, Stacy A, Fleming D *et al.* Co-infecting microorganisms dramatically alter pathogen gene essentiality during polymicrobial infection. *Nat Microbiol* 2017;**2**(8):17079. https://doi.org/10.1038/nmicrobiol.2017.79.

4. Ibberson CB, Barraza JP, Holmes AL *et al.* Precise spatial structure impacts antimicrobial susceptibility of S. aureus in polymicrobial wound infections. *Proc Natl Acad Sci U S A* 2022;**119**(51):e2212340119. https://doi.org/10.1073/pnas.2212340119.

5. Turner KH, Everett J, Trivedi U *et al.* Requirements for Pseudomonas aeruginosa Acute Burn and Chronic Surgical Wound Infection. *PLOS Genet* 2014;**10**(7):e1004518. https://doi.org/10.1371/journal.pgen.1004518.

6. Watters C, DeLeon K, Trivedi U *et al.* Pseudomonas aeruginosa biofilms perturb wound resolution and antibiotic tolerance in diabetic mice. *Med Microbiol Immunol (Berl)* 2013;**202**(2):131–41. https://doi.org/10.1007/s00430-012-0277-7.

7. Cornforth DM, Dees JL, Ibberson CB *et al.* Pseudomonas aeruginosa transcriptome during human infection. *Proc Natl Acad Sci* 2018;**115**(22):E5125–34. https://doi.org/10.1073/pnas.1717525115.

8. Fritz BG, Kirkegaard JB, Nielsen CH *et al.* Transcriptomic fingerprint of bacterial infection in lower extremity ulcers. *Apmis* 2022;**130**(8):524–34. https://doi.org/10.1111/apm.13234.

9. Malone M, Radzieta M, Peters TJ *et al.* Host-microbe metatranscriptome reveals differences between acute and chronic infections in diabetes-related foot ulcers. *APMIS* 2022;**130**(12):751–62. https://doi.org/10.1111/apm.13200.

10. Kalan LR, Meisel JS, Loesche MA *et al.* Strain- and Species-Level Variation in the Microbiome of Diabetic Wounds Is Associated with Clinical Outcomes and Therapeutic Efficacy. *Cell Host Microbe* 2019;**25**(5):641-655.e5. https://doi.org/10.1016/j.chom.2019.03.006.

11. Radzieta M, Sadeghpour-Heravi F, Peters TJ *et al.* A multiomics approach to identify host-microbe alterations associated with infection severity in diabetic foot infections: a pilot study. *Npj Biofilms Microbiomes* 2021;**7**(1):1–12. https://doi.org/10.1038/s41522-021-00202-x.

12. LaMar D. *FastQC*. published online 2015. https://doi.org/https://qubeshub.org/resources/fastqc.

13. Martin M. Cutadapt removes adapter sequences from high-throughput sequencing reads. *EMBnetJournal* 2011;**17**(1):art. 1. https://doi.org/10.14806/ej.17.1.200.

14. Prjibelski A, Antipov D, Meleshko D *et al.* Using SPAdes De Novo Assembler. *Curr Protoc Bioinforma* 2020;**70**(1):e102. https://doi.org/10.1002/cpbi.102.

15. Langmead B, Salzberg SL. Fast gapped-read alignment with Bowtie 2. *Nat Methods* 2012;**9**(4):357–9. https://doi.org/10.1038/nmeth.1923.

16. Kopylova E, Noé L, Touzet H. SortMeRNA: fast and accurate filtering of ribosomal RNAs in metatranscriptomic data. *Bioinformatics* 2012;**28**(24):3211–7. https://doi.org/10.1093/bioinformatics/bts611.

17. Blanco-Míguez A, Beghini F, Cumbo F *et al.* Extending and improving metagenomic taxonomic profiling with uncharacterized species using MetaPhlAn 4. *Nat Biotechnol* 2023;**41**(11):1633–44. https://doi.org/10.1038/s41587-023-01688-w.

18. Beghini F, McIver LJ, Blanco-Míguez A *et al.* Integrating taxonomic, functional, and strain-level profiling of diverse microbial communities with bioBakery 3. *eLife* 2021;**10**:e65088. https://doi.org/10.7554/eLife.65088.

19. Vallenet D, Labarre L, Rouy Z *et al.* MaGe: a microbial genome annotation system supported by synteny results. *Nucleic Acids Res* 2006;**34**(1):53–65. https://doi.org/10.1093/nar/gkj406.

20. Cornforth DM, Diggle FL, Melvin JA *et al.* Quantitative Framework for Model Evaluation in Microbiology Research Using Pseudomonas aeruginosa and Cystic Fibrosis Infection as a Test Case. *mBio* 2020;**11**(1):e03042-19. https://doi.org/10.1128/mBio.03042-19.

21. Lewin GR, Stocke KS, Lamont RJ *et al.* A quantitative framework reveals traditional laboratory growth is a highly accurate model of human oral infection. *Proc Natl Acad Sci* 2022;**119**(2):e2116637119. https://doi.org/10.1073/pnas.2116637119.

22. Lewin GR, Kapur A, Cornforth DM *et al.* Application of a quantitative framework to improve the accuracy of a bacterial infection model. *Proc Natl Acad Sci* 2023;**120**(19):e2221542120. https://doi.org/10.1073/pnas.2221542120.

23. Boles BR, Thoendel M, Roth AJ *et al.* Identification of Genes Involved in Polysaccharide-Independent Staphylococcus aureus Biofilm Formation. *PLoS ONE* 2010;**5**(4):e10146. https://doi.org/10.1371/journal.pone.0010146.

24. Stover CK, Pham XQ, Erwin AL *et al.* Complete genome sequence of Pseudomonas aeruginosa PAO1, an opportunistic pathogen. *Nature* 2000;**406**(6799):959–64. https://doi.org/10.1038/35023079.

25. Sahm DF, Kissinger J, Gilmore MS *et al.* In vitro susceptibility studies of vancomycin-resistant Enterococcus faecalis. *Antimicrob Agents Chemother* 1989;**33**(9):1588–91. https://doi.org/10.1128/AAC.33.9.1588.

26. Madoff LC, Michel JL, Kasper DL. A monoclonal antibody identifies a protective C-protein alpha-antigen epitope in group B streptococci. *Infect Immun* 1991;**59**(1):204–10. https://doi.org/10.1128/iai.59.1.204-210.1991.

27. Dalton T, Dowd SE, Wolcott RD *et al.* An In Vivo Polymicrobial Biofilm Wound Infection Model to Study Interspecies Interactions. *PLoS ONE* 2011;**6**(11):e27317. https://doi.org/10.1371/journal.pone.0027317.

28. Fujii M, Mizutani Y, Sakuma T *et al.* *Corynebacterium kroppenstedtii* in granulomatous mastitis: Analysis of formalin‐fixed, paraffin‐embedded biopsy specimens by immunostaining using low‐specificity bacterial antisera and real‐time polymerase chain reaction. *Pathol Int* 2018;**68**(7):409–18. https://doi.org/10.1111/pin.12683.

**Dataset S1 (separate file):** Distribution of functions for each annotation scheme in each sample. **A**: Column A to G showing all the human samples and their associated number of features from different annotations; Column J showing the 6 human samples that were removed prior to inflection point analysis. **B**: Showing emphasis on samples whose UniRef IDs were lesser than GO terms when collapsed. **C**: Showing the inflection point analysis result and how many IDs make 99%. **D**: Showing the distribution of the COG IDs after inflection point analysis. **E**: Showing the distribution of COG IDs in all the samples with their mean.

**Dataset 2 (separate file):** Taxonomic data from MetaPhlAn4 for metatranscriptomic and metagenomic datasets. Cumulative abundance data of species in the metatranscriptomic (**A**) and metagenomic (**B**) datasets also showing the top 80% and overlaps between the two datasets (**C**).

**Dataset 3 (separate file):** Metabolism related COG IDs (**A-C**) outside the AS range (-2 and +2) in the S. aureus mono-culture but inside this range for the 6-Member with (**A**) showing the COG IDs and their Zscores, (**B**) showing their stratified contribution and (**C**) showing their product annotations. (D-F) within the AS range (-2 and +2) in both the S. aureus mono-culture and the 6-Member with (**D**) showing the COG IDs and their Zscores, (**E**) showing their stratified contribution and (**F**) showing their species related annotations.

**Supplementary figures**

**
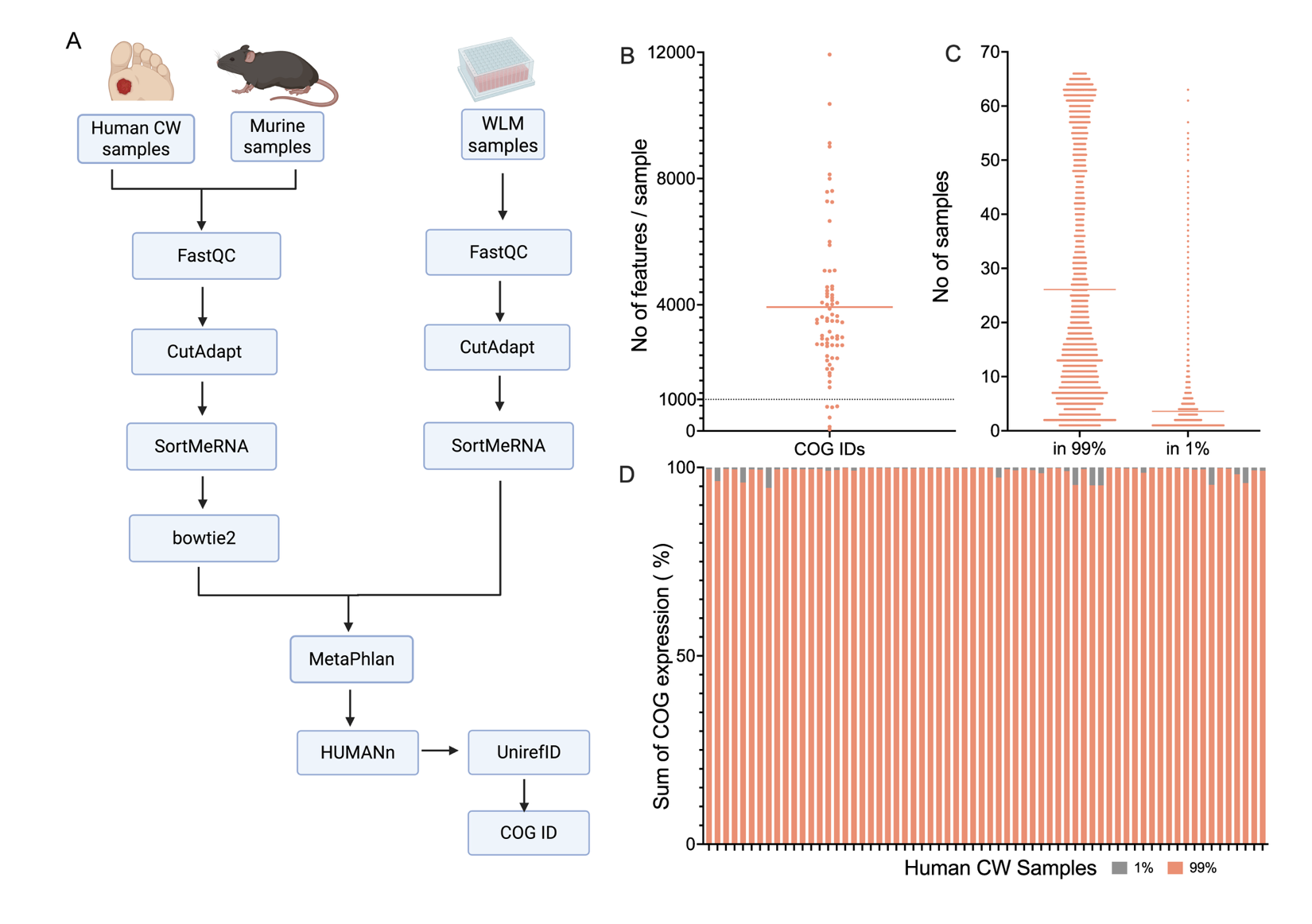
**

**Figure S1. Pipeline and quality control for inclusion of COG IDs in analysis pipeline. (A)** Graphical representation of bioinformatic steps for sample preprocessing and downstream analysis. Image was generated with biorender. **(B)** Distribution of COG IDs in human CW metatranscriptomic samples before inflection point analysis. Line at 1000 to highlight samples with less than 1000 COG IDs. **(C)** Representation of the number of samples that possess the COG IDs that make up the 99% (included in framework) and 1% (not included in framework) of expression data. Line at mean 26 and 4 for the 99% and 1% data, respectively. **(D)** How the COG IDs that make up the 99% and 1% of entire expression are distributed across the human CW samples.

**
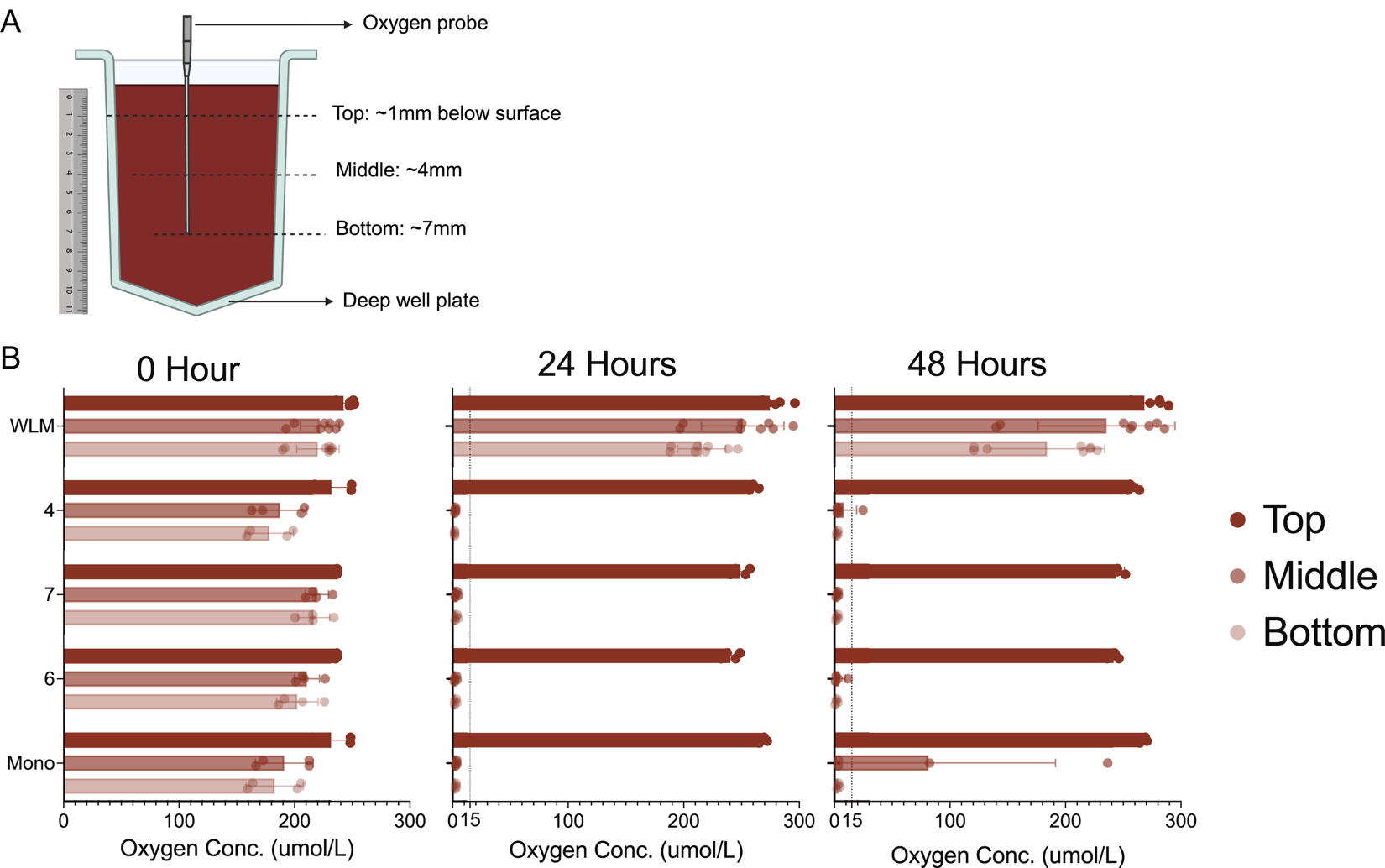
**

**Figure S2**: **Stratification of oxygen levels is present in the in vitro experimental models**. **(A)** Diagram showing culture volume (~11mm from bottom - ruler). We measured oxygen concentration (µmol/L) across three depths from surface (~1mm – top; ~4mm – middle; ~7mm - bottom) using the Unisense microprofiling system with an oxygen microsensor 25µM probe (OX-25). Image was generated with biorender. **(B)** Oxygen concentration in the WLM culture conditions falls to <15µmol/L at 24 and 48 hours post inoculation.

**
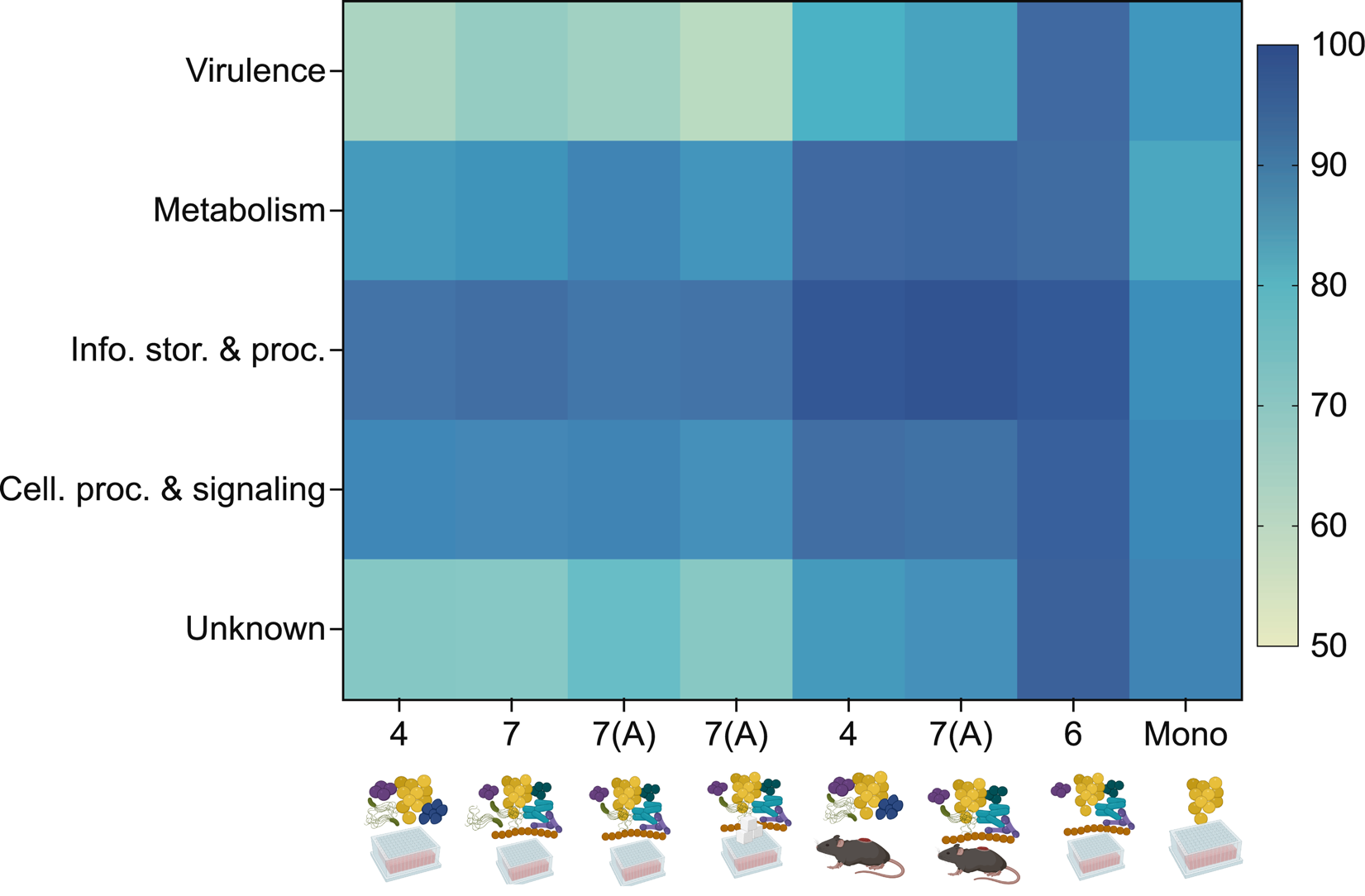
**

**Figure S3:** **Virulence and cellular processes are highly impacted categories in the model communities.** A heat map showing the distribution of the accuracy scores of the main categories in the samples evaluated with the overall accuracy scores being 4member_wlm 77.98%, 7member_wlm 78.00%, 7member_altered_wlm 82.02%, 7member_altered_hyperglycemia 77.62%, 4member_murine 89.70%, 6member_wlm 96.27%, *S. aureus* monoculture 89.80%. Abbreviations: 4 = 4member, 7 = 7member, 7(A) = 7member with altered inoculum, 6 = 6member, Mono = *S. aureus* monoculture in any condition. Graphical annotations were generated with biorender.

**
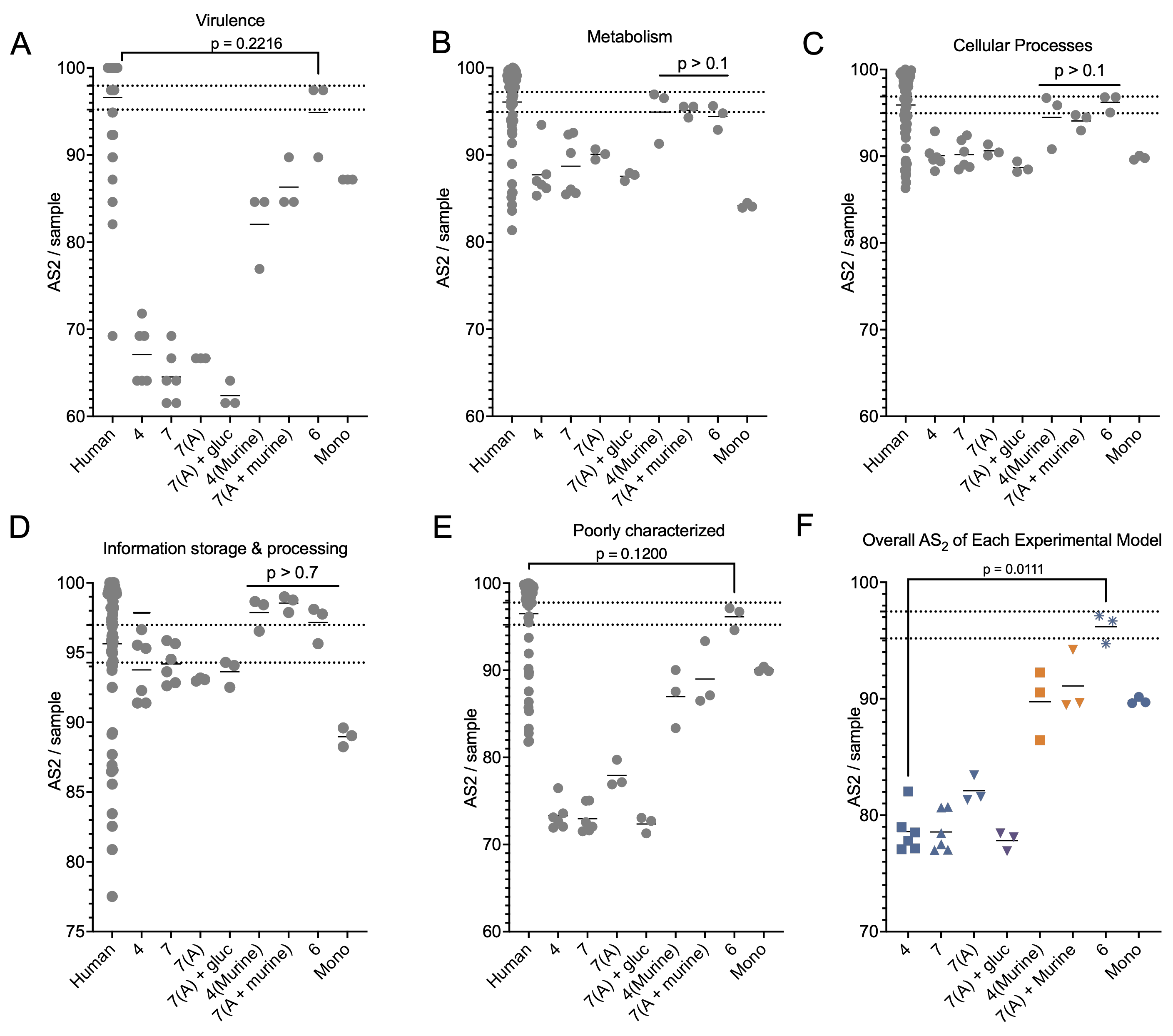
**

**Figure S4: Statistical significance of the experimental models. A-E** compare the models to the human samples in the **(A)** Virulence category. Only the 6-member WLM community is not significantly different from the human CW samples (p-value = 0.2216 Mann-Whitney test). Dotted lines on 95% CI of human CW AS2 mean (Lower = 95.21; Upper = 97.95). **(B)** Metabolism category. The 4-murine, 7-member murine and 6-member WLM community are not significantly different from the human CW samples (each p-value > 0.1 Mann-Whitney test). Dotted lines on 95% CI of human CW AS2 mean (Lower = 94.9; Upper = 97.2). (**C**) Cellular processes. The 4-murine, 7-member murine and 6-member WLM community are not significantly different from the human CW samples (each p-value > 0.1 Mann-Whitney test). Dotted lines on 95% CI of human CW AS2 mean (Lower = 94.96; Upper = 96.88).  **(D)** Information storage and processing. The 4-murine, 7-member murine and 6-member WLM community are not significantly different from the human CW samples (each p-value > 0.7 Mann-Whitney test). Dotted lines on 95% CI of human CW AS2 mean (Lower = 94.28; Upper = 96.98). **(E)** Poorly characterized category. Only the 6-member WLM community is not significantly different from the human CW samples (p-value = 0.1200 Mann-Whitney test). Dotted lines on 95% CI of human CW AS2 mean (Lower = 95.23; Upper = 97.77). **(F)** The improved experimental models compared to the initial 4-member WLM samples. Only the 6-member WLM model was significantly different from the initial 4-member WLM sample (p = 0.011, Dotted lines on 95% CI of human CW AS2 mean (Lower = 95.18; Upper = 97.49, Kruskal-Wallis and Dunn’s multiple comparison tests). Model samples are shaped by community structure and colored by infection environment. Purple – WLM, Orange - Murine.

**
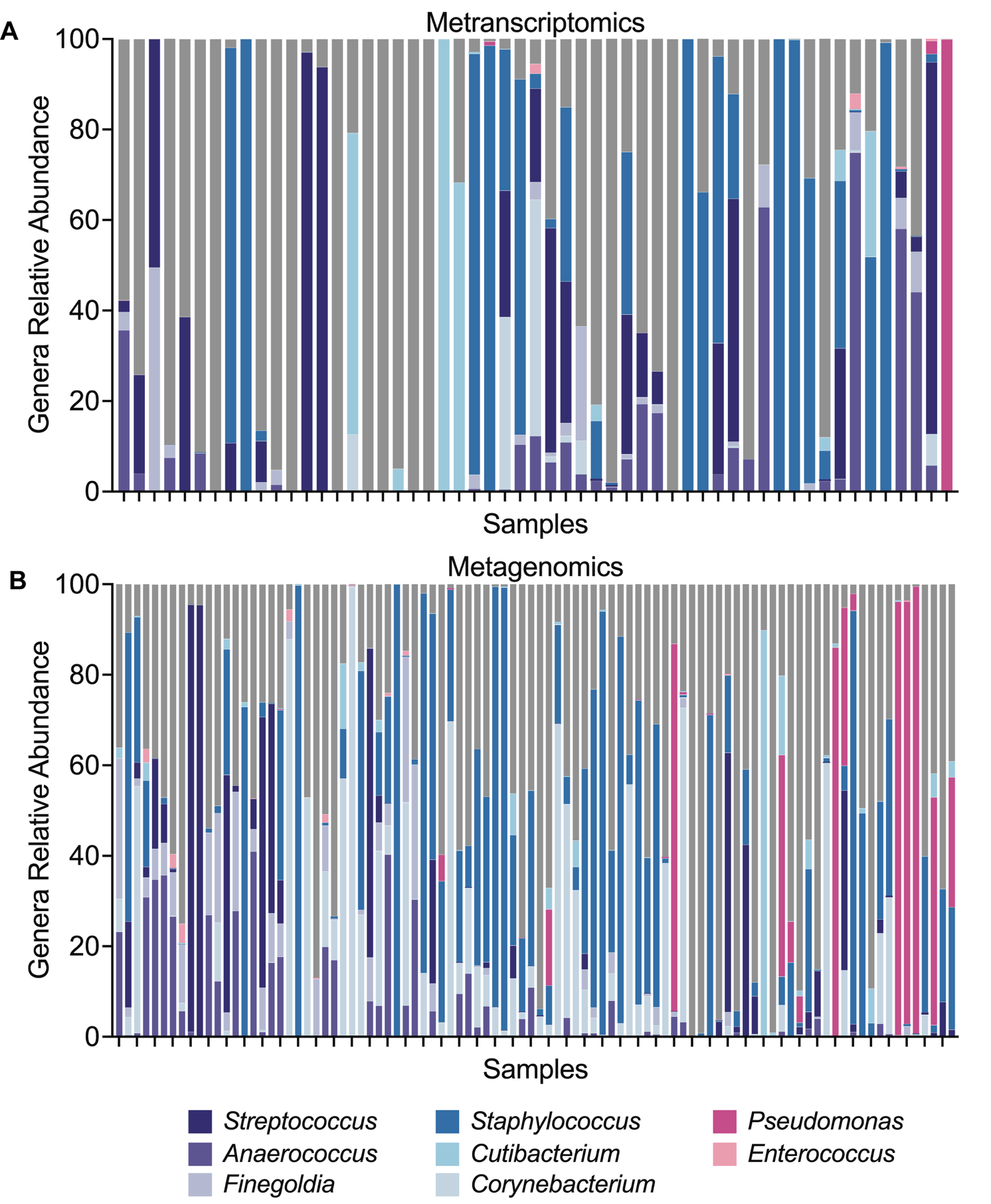
**

**Figure S5**: **Members of the improved mock communities are present in the human CW samples.** A&B Omics data from (**A**) metatranscriptomic and (**B**) metagenomic datasets with community composition highlighting the members of the mock communities tested in our models.

**
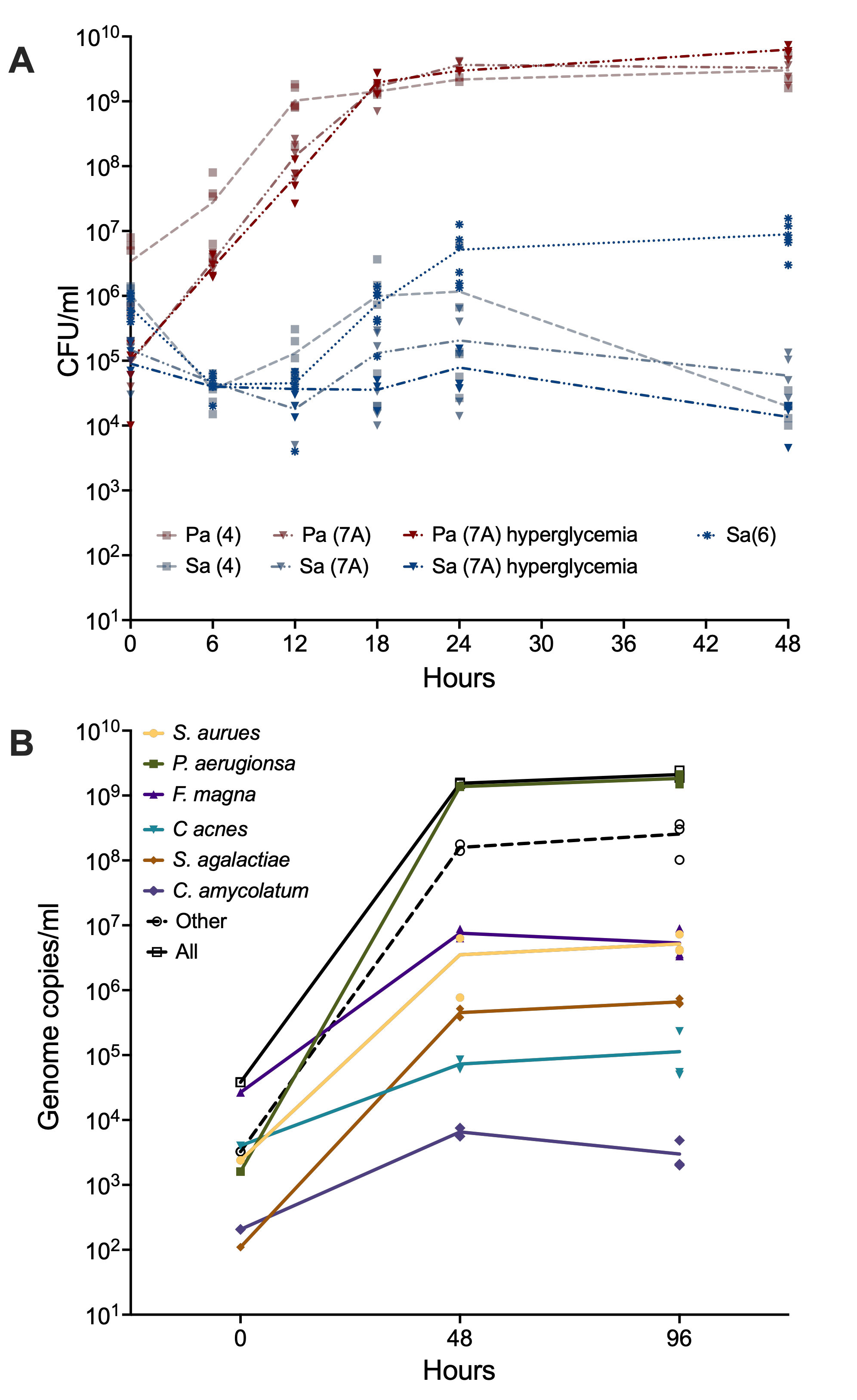
**

**Figure S6: Growth of bacteria species in laboratory models. (A)** Increase in the burden of *P. aeruginosa* shows a decrease in the burden of *S. aureus* in co-cultures. Graph shows the growth curve in WLM and hyperglycemic WLM to specifically track the growth of *S. aureus* (Sa) (blue) and *P. aeruginosa* (Pa) (red) in the four, six and seven member communities. **(B)** qPCR results to show bacterial burden in the 7member community with altered inoculum in WLM.

**
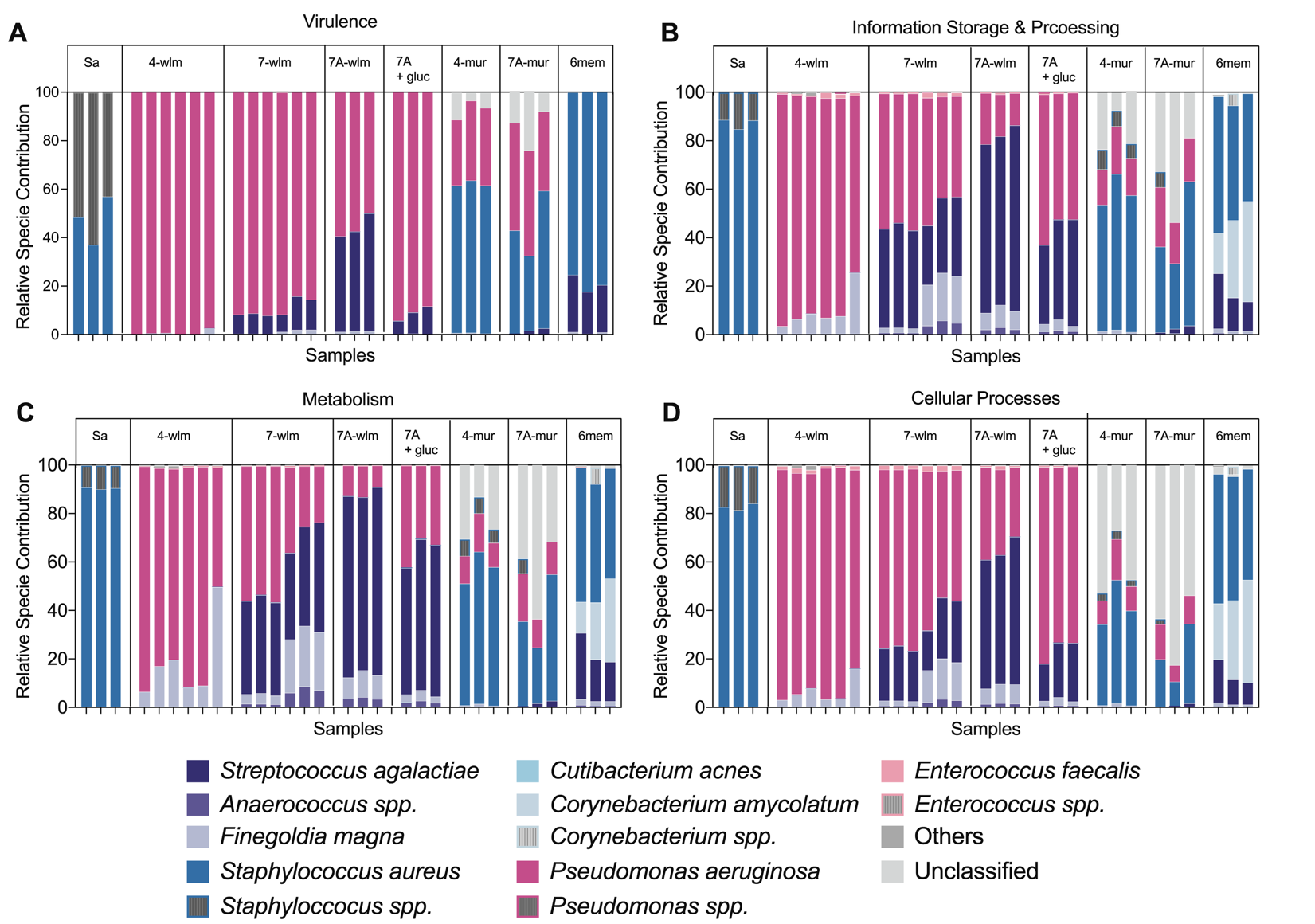
**

**Figure S7: *P. aeruginosa* drive the expression of community functions in less accurate models.** Community contribution in all the experimental models tested in the (**A**) Virulence (**B**) Information storage and processing (**C**) Metabolism and (**D**) Cellular processes categories. The stratified functions from HUMANn4 were analyzed to obtain the cumulative CPM expression of all the COG IDs in each meta category. For each category, the relative species contribution equates the cumulative contribution of each species to the cumulative CPM in that category.

**
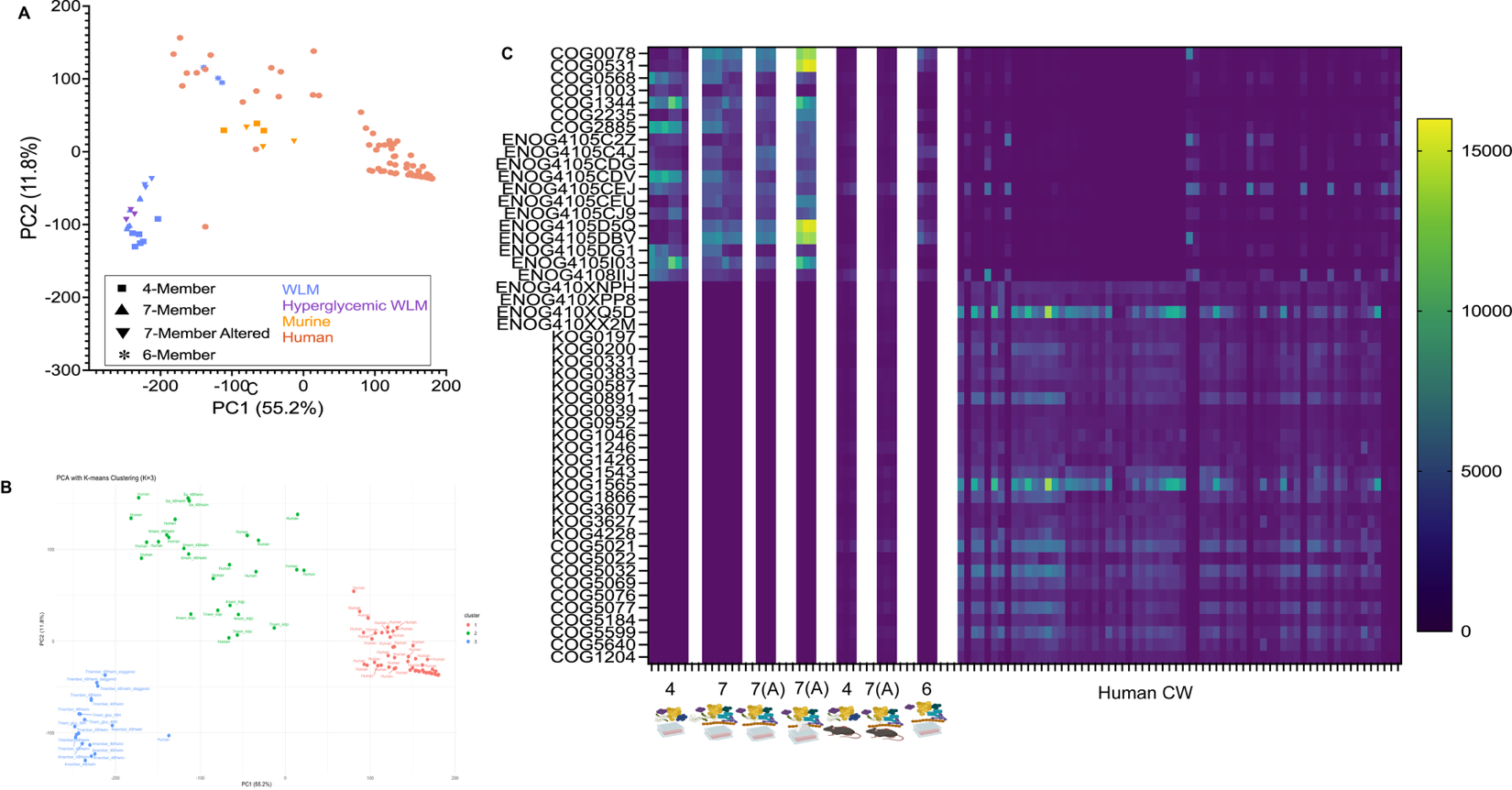
**

**Figure S8: Six-member community (in vitro) and the in vivo four- and seven- member communities cluster more closely to some human samples.** (A). PCA to show the clustering of samples. Samples are grouped as humans or model. Model samples are shaped by community structure and colored by infection environment. **(B)** Highlighting the clustering similarity using k-means clustering (**C)** Top 50 COG IDs that drive the clustering are expressed in similar patterns across the human, murine and invitro six member samples. Abbreviations: 4 = 4member, 7 = 7member, 7(A) = 7member with altered inoculum, 6 = 6member, Mono = *S. aureus* monoculture in any condition. Graphical annotations were generated with biorender.

**
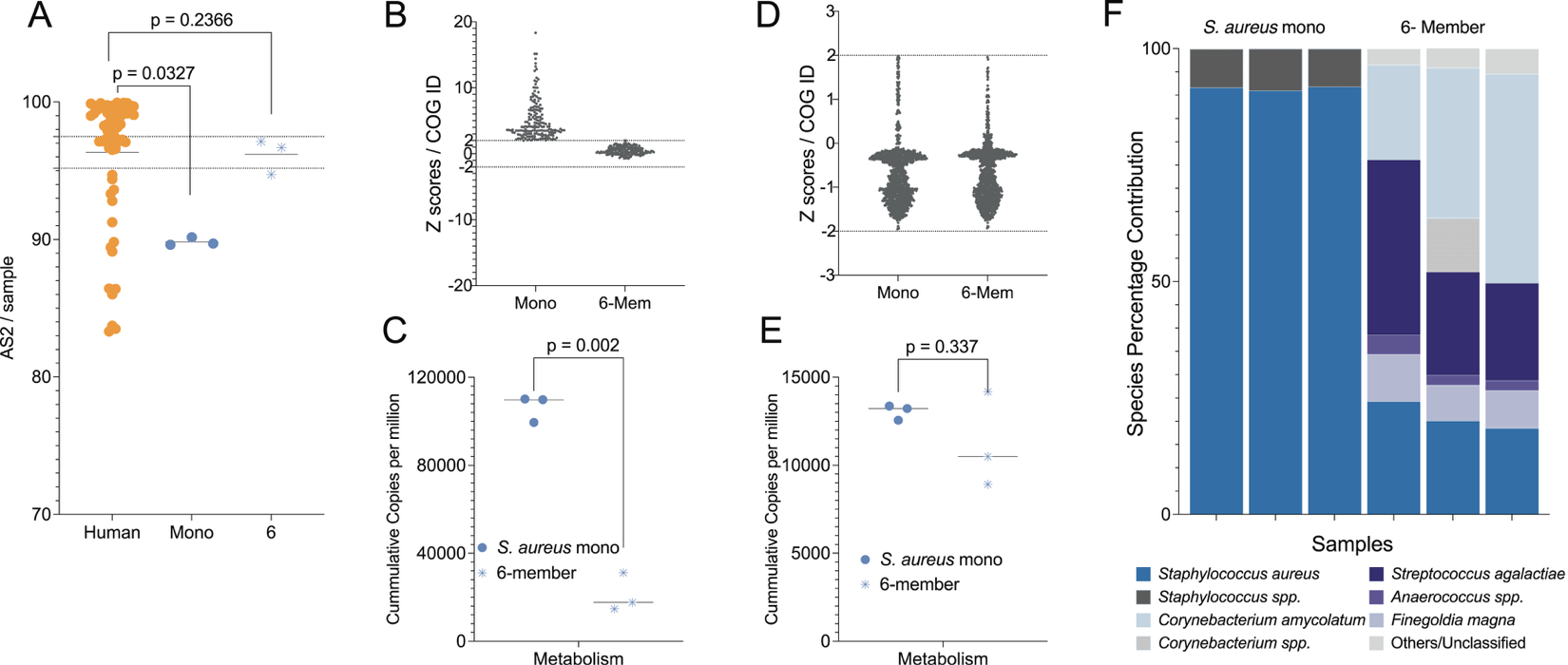
**

**Figure S9. The 6-member community better captures in situ microbial physiology and it is not solely driven by *S. aureus*. (A)** Graph showing the distribution of the 6-member and S. aureus mono-culture models as they compare to the human CW samples. The AS2 of the 6-member model is within the variation observed in the human samples (Dotted lines on 95% CI of human CW AS2 mean (Lower = 95.18; Upper = 97.49). Also, the model is not significantly different from human samples (p = 0.2366, Mann-Whitney test) while the S. aureus mono-culture is outside of the human variation and significantly different (p = 0.0327, Mann-Whitney test). **B&C** Metabolism-associated COGs whose Z scores are outside the AS2 range (Z-score −2 to +2) in *S. aureus* monoculture but within this range in the six-member community. (**B**) Distribution of the Z scores of those COG IDs (**C**) Sum of expression in CPM of the COG IDs identified in B (p = 0.002, Mann-Whitney test). **D-F** Metabolism-associated COGs whose Z scores are within the AS2 range (Z-score −2 to +2) in both the *S. aureus* monoculture and the six-member community. (**D**) Distribution of the Z scores of those COG IDs (**E**) Sum of expression in CPM of the COG IDs identified in D (p = 0.337, Mann-Whitney test). (**F**) Percentage of the contribution of the individual species in each community to the summed expression shown in E.

**
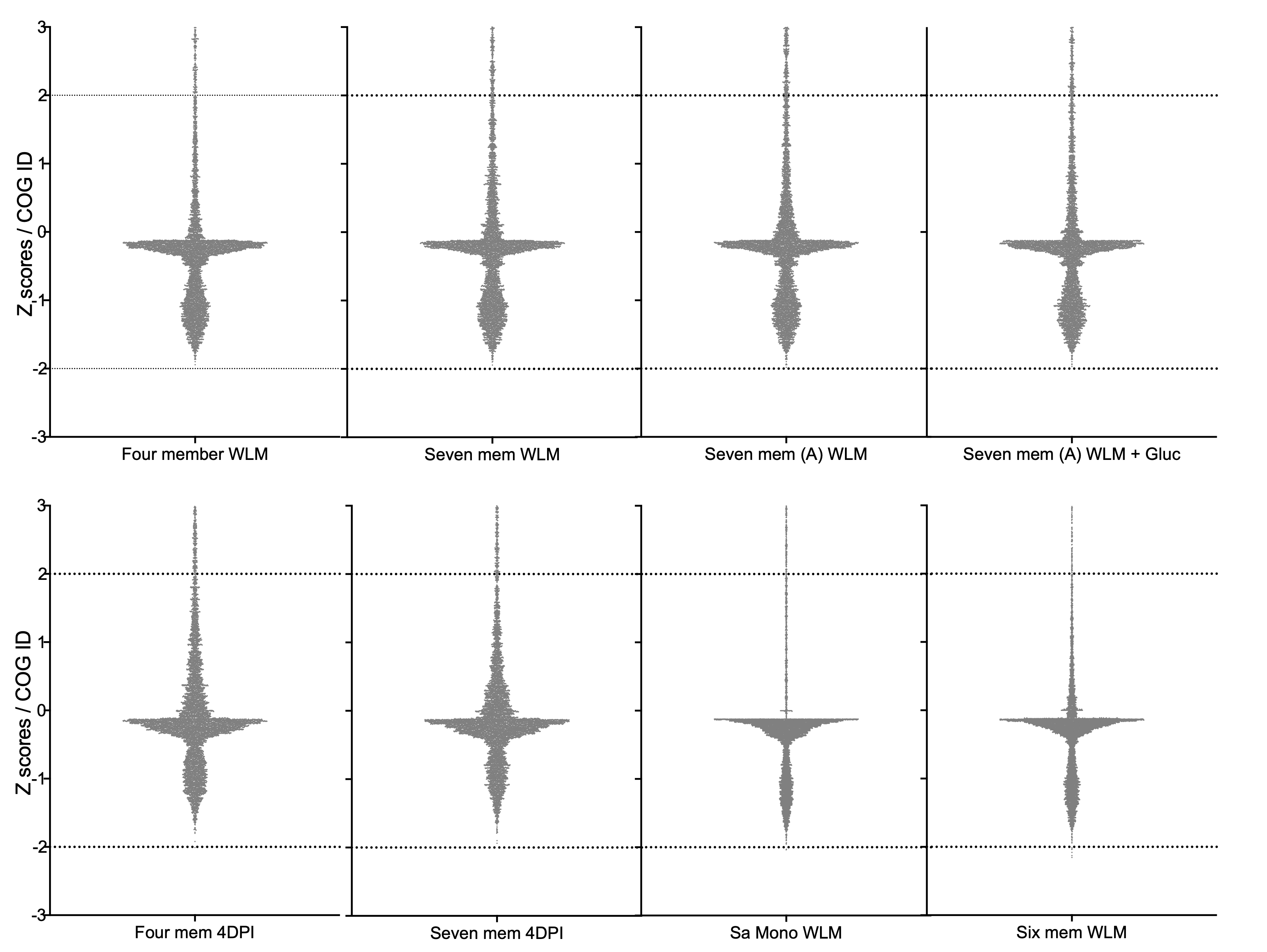
**

**Figure S10:** Distribution of the Z scores that fall within -3 and +3 to highlight those within the AS2 range (-2 and +2) in all the experimental models tested. Abbreviations: Seven mem (A) = 7member with altered inoculum, Seven mem (A) WLM + Gluc = 7member with altered inoculum in the hyperglycemic WLM,
